# Atopic Dermatitis-Like Disease and Associated Lethal Myeloproliferative Disorder Arise from Loss of Notch Signaling in the Murine Skin

**DOI:** 10.1371/journal.pone.0009258

**Published:** 2010-02-18

**Authors:** Alexis Dumortier, André-Dante Durham, Matteo Di Piazza, Sophie Vauclair, Ute Koch, Gisèle Ferrand, Isabel Ferrero, Shadmehr Demehri, Lynda Li Song, Andrew G. Farr, Warren J. Leonard, Raphael Kopan, Lucio Miele, Daniel Hohl, Daniela Finke, Freddy Radtke

**Affiliations:** 1 Ecole Polytechnique Fédérale de Lausanne (EPFL SV ISREC), Lausanne, Switzerland; 2 Ludwig Institute for Cancer Research, Lausanne Branch, University of Lausanne, Epalinges, Switzerland; 3 Department of Developmental Biology and Division of Dermatology, Washington University School of Medicine, St. Louis, Missouri, United States of America; 4 Breast Cancer Program, Cardinal Bernardin Cancer Center, Loyola University Chicago, Chicago, Illinois, United States of America; 5 Department of Biological Structure and Department of Immunology, University of Washington, Seattle, Washington, United States of America; 6 Laboratory of Molecular Immunology, National Heart, Lung, and Blood Institute, National Institutes of Health, Bethesda, Maryland, United States of America; 7 Department of Dermatology, Centre Hospitalier Universitaire Vaudois, Lausanne, Switzerland; 8 Center for Biomedicine, Department of Clinical and Biological Sciences (DKBW), University of Basel, Basel, Switzerland; Centre de Recherche Public de la Santé (CRP-Santé), Luxembourg

## Abstract

**Background:**

The Notch pathway is essential for proper epidermal differentiation during embryonic skin development. Moreover, skin specific loss of Notch signaling in the embryo results in skin barrier defects accompanied by a B-lymphoproliferative disease. However, much less is known about the consequences of loss of Notch signaling after birth.

**Methodology and Principal Findings:**

To study the function of Notch signaling in the skin of adult mice, we made use of a series of conditional gene targeted mice that allow inactivation of several components of the Notch signaling pathway specifically in the skin. We demonstrate that skin-specific inactivation of Notch1 and Notch2 simultaneously, or RBP-J, induces the development of a severe form of atopic dermatitis (AD), characterized by acanthosis, spongiosis and hyperkeratosis, as well as a massive dermal infiltration of eosinophils and mast cells. Likewise, patients suffering from AD, but not psoriasis or lichen planus, have a marked reduction of Notch receptor expression in the skin. Loss of Notch in keratinocytes induces the production of thymic stromal lymphopoietin (TSLP), a cytokine deeply implicated in the pathogenesis of AD. The AD-like associated inflammation is accompanied by a myeloproliferative disorder (MPD) characterized by an increase in immature myeloid populations in the bone marrow and spleen. Transplantation studies revealed that the MPD is cell non-autonomous and caused by dramatic microenvironmental alterations. Genetic studies demontrated that G-CSF mediates the MPD as well as changes in the bone marrow microenvironment leading to osteopenia.

**Significance:**

Our data demonstrate a critical role for Notch in repressing TSLP production in keratinocytes, thereby maintaining integrity of the skin and the hematopoietic system.

## Introduction

The skin epidermis and its appendages represent a constantly renewing physical barrier that protects against mechanical injuries, infective organisms and excessive loss of water [1]. Cellular processes such as proliferation, migration and cell death must be highly regulated in order to ensure life long homeostasis. The molecular pathways controlling these processes have only just started to be explored. The Notch pathway plays a key role in differentiation of the epidermis and its appendages. Notch proteins comprise a family of four type I transmembrane receptors that influence cell fate decision and differentiation processes in multiple organisms and tissues [2], [3]. Notch signaling is triggered upon the binding of ligands of the Jagged and Delta family, which leads to the proteolytic release of the intracellular cytoplasmic domain of Notch receptors (NIC). The released NIC subsequently translocates to the nucleus where it binds to the transcriptional repressor CSL (RBP-J) to activate target gene expression [4].

Most of our current knowledge regarding Notch signaling in skin and hair follicles is derived from both gain and loss of function studies in primary keratinocytes, or from genetic studies inactivating different Notch signaling components during embryonic development [5], [6], [7], [8], [9], [10], [11], [12], [13]. Thus, Notch mediated RBP-J signaling has been shown to be important for terminal differentiation and maintenance of hair follicles and sebaceous glands [6], [7], [14]. Moreover, Notch signaling was shown to be important in the specification of the spinous layer through the Notch target gene *Hes1* and in the downregulation of the basal cell fate of the interfollicular epidermis [5], [10], [12]. Skin-specific ablation of Notch signaling during embryogenesis leads to death before weaning, due to the loss of epidermal barrier function accompanied by the development of a systemic B-lymphoproliferative disorder (B-LPD) [13].

The role of Notch signaling in adult skin is less clear and has only been partially investigated [5], [10], [14], [15]. In postnatal skin, Notch signaling is predominantly mediated through the Notch1 and Notch2 receptors [5]. Postnatal K5-Cre^ERT^ mediated inactivation of Notch1 in the skin results in hyperproliferation with expansion of the proliferative basal cell layer, hair loss and epidermal cyst formation within less then one month [5], [14]. A long-term consequence of Notch1 deficiency in adult skin is the development of skin tumors, suggesting that in addition to regulating differentiation processes in the skin, Notch signaling is also associated with tumor suppressive functions [15], [16].

Here, we show that simultaneous ablation of Notch1 and Notch2 signaling in the adult skin results in a severe form of atopic dermatitis-like disease as a result of highly elevated levels of TSLP. The AD-like disease is accompanied by a cell non-autonomous G-CSF induced myeloproliferative disorder and osteopenia, all of which is caused by TSLPR mediated signaling.

## Results

### Postnatal Loss of Notch Signaling in the Skin Results in an Atopic Dermatitis (AD)-Like Disease in the Adult Mouse

**Figure 1:**
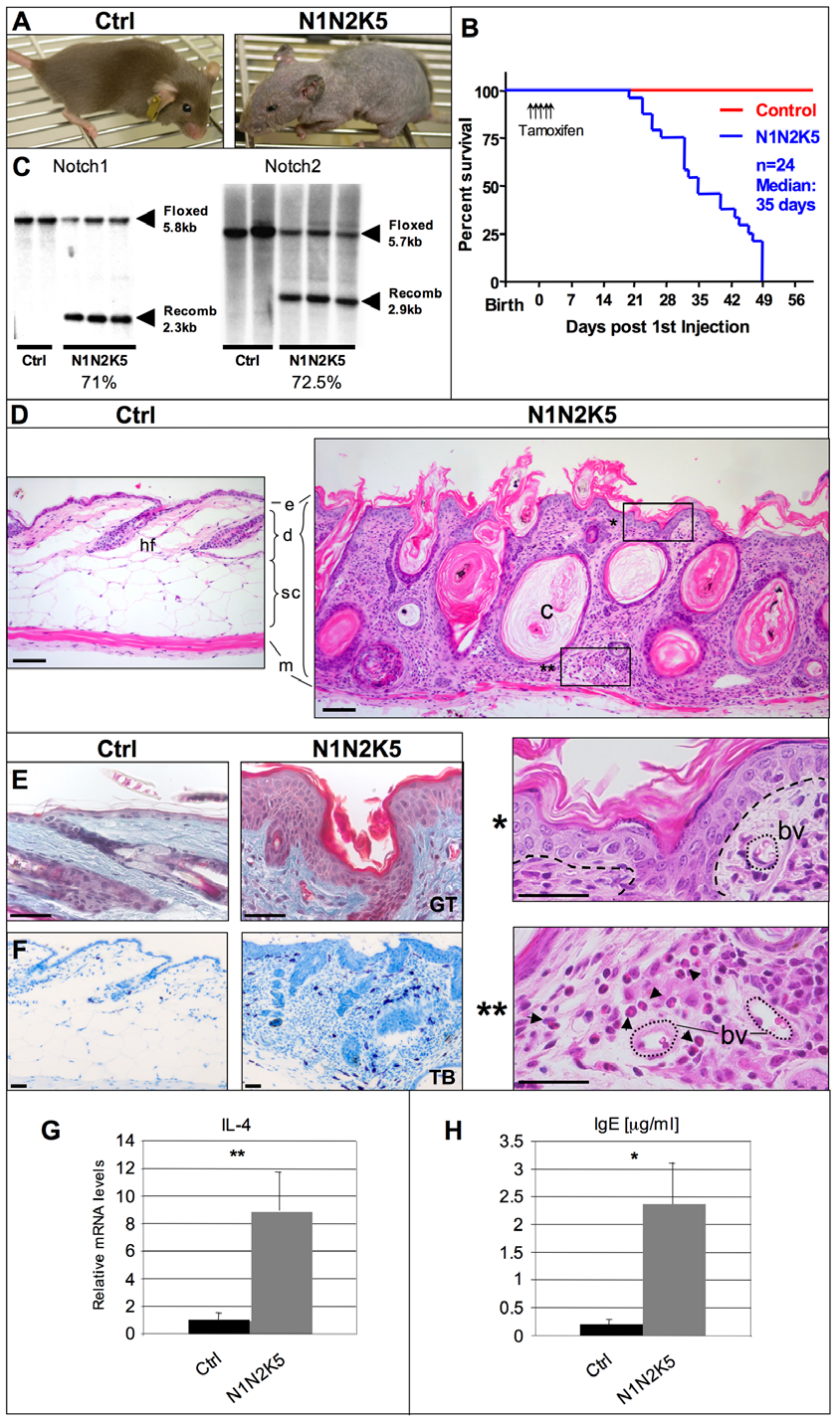
Loss of Notch signaling in post-natal epidermis leads to a severe form of atopic dermatitis and lethality. (**A**) Representative photograph of Control (Ctrl) and N1N2K5 mice 38 days post first injection of tamoxifen showing loss of hair, thick, dry, and scaly skin. (**B**) Survival curve of control (Ctrl, n = 20) and N1N2K5 (n = 24) mice after tamoxifen injection. The survival curve is the combined result of 3 individual experiments. (**C**) Southern blot analysis of genomic DNA from scraped epidermis from control (Ctrl, n = 2) and N1N2K5 mice (n = 3) showing the floxed and the recombined (Recomb) alleles of Notch1 and Notch2 respectively. The recombination efficiency is >70% for both genes. Three individual experiments were performed. (**D**) Representative HE staining on control (Ctrl) and N1N2K5 dorsal skin sections showing a thickened epidermal layer (e) a massively infiltrated dermis (d) with large epidermoid cysts (c) from degenerated hair follicles (hf) and absence of subcutis (sc) above the muscles (m). Asterisks indicate enlarged regions of the skin showing acanthosis, hyperkeratosis and spongiosis of the epidermis as well as eosinophil infiltrates (arrows) around dilated blood vessels (bv) in the dermis (n = 8, 4 individual experiments were performed). (**E**) Goldner's Trichrome (GT) readily shows the spongiosis and hyperkeratosis (n = 7, 4 individual experiments were performed). (**F**) Toluidine blue (TB) staining on control (Ctrl) and N1N2K5 skin sections showing massive infiltration of mast cells (dark blue) (n = 7, 4 individual experiments were performed). (**G**) Quantitative RT-PCR on dermis-derived RNA for the T helper specific cytokine IL-4 from Ctrl and N1N2K5 mice. The experiment was performed in triplicates (n = 3 per sample group, two individual experiments were performed). (**H**) A 16-fold increase in serum IgE levels is observed in N1N2K5 compared to Ctrl mice. The experiment was performed in triplicates (n = 3 per sample group, three individual experiments). (* p<0.01; ** p<0.001). [Scale bars: 50 µm].

The function of Notch signaling during skin homeostasis was characterized using mice bearing homozygously floxed alleles for *Notch1*, for *Notch2*, for both *Notch1* and *Notch2* [17], and for *RBP-J* [18]. These mice were crossed to transgenic mice expressing a tamoxifen inducible Cre-recombinase (Cre^ERT^) under the control of the *Keratin*5 promoter [19] (hereafter: N1K5, N2K5, N1N2K5, RBP-JK5). Eight day-old mutant mice and corresponding littermate controls, lacking the Cre^ERT^ transgene, were injected with tamoxifen for 5 consecutive days and analyzed 30 to 40 days post injection (Figure 1). Gene specific deletion efficiency was assessed by Southern blot analysis of genomic DNA isolated from the epidermis of the different gene targeted mice at the time point of analysis. The recombination efficiency in N1K5 and N2K5 was >80% (data not shown), and 70% in N1N2K5 mice for both the Notch1 and the Notch2 genes (Figure 1C). Postnatal skin specific inactivation of Notch1 resulted in loss of skin appendages, deregulation of several epidermal differentiation markers and hyperproliferation (Figure S1) as previously reported [5]. In contrast, postnatal skin-specific loss of Notch2 did not lead to any apparent phenotype (Figure S1), which is similar to data describing embryonic inactivation of Notch2 [7]. This suggests that either Notch2 has no function in murine skin, or that its loss is fully compensated by redundant Notch1 signaling. This question was addressed by analyzing mice with simultaneous inactivation of both Notch1 and Notch2 in the epidermis. Moreover, the phenotype of these mice was compared to mice in which RBP-J, the downstream mediator for all Notch receptors, was inactivated.

N1N2K5 mice start to lose hair as soon as 1 week post tamoxifen injection and show complete and irreversible hair loss after 1 month (Figure 1A). Interestingly, mice with skin specific inactivation of both Notch1 and Notch2 receptors, or RBP-J, but not of individual receptors, became moribund and died with 100% penetrance within 7 weeks of tamoxifen injection (Figure 1B and data not shown).

Histological analysis of Notch mutant mice revealed a massive dermal hypercellularity, a complete loss of subcutaneous fat, the presence of numerous hyperproliferative epidermoid cysts in the dermis and a thickened and hyperkeratinized epidermis (acanthosis and hyperkeratosis) (Figure 1D, E and Figure S1) in mutant skin sections of N1N2K5 and RBP-JK5, but not of N1K5 or N2K5 mice. In addition, the epidermis of N1N2K5 (Figure 1D, E) and RBP-JK5 (data not shown) mice showed signs of intercellular oedema (epidermal spongiosis). The underlying dermis exhibited inflammatory infiltrates, mostly characterized by eosinophils, the accumulation of mast cells, and dilated blood vessels (Figure 1D, F). Moreover, flow cytometric analysis of dermis derived CD45^+^ cells revealed an increase in the percentage of these cells as well as in inflammatory (CD11c^+^CD11b^+^Ly6C^+^) dendritic cells (DC) [20], CD103^+^ Langerin^+^ DC [21], T and B cells as well as neutrophiles in N1N2K5 mice as compared to control animals (Figure S2). Quantitative real-time PCR for T helper specific cytokines using dermis-derived RNA revealed increased IL-4 and IL-13 mRNA levels in *Notch* mutant mice, whereas mRNA levels for IFNγ, IL-12, IL-17a, IL-21 and IL-22 where not significantly altered, indicating an infiltration of Th2 cells (Figure 1G, and Figure S3). Interestingly, we also found high serum IgE levels in the *Notch* mutant mice (Figure 1H). Taken together, these results suggest that loss of Notch signaling in the epidermis of adult mice induces a strong inflammatory response with many hallmarks of Atopic Dermatitis (AD; referred hereafter as AD-like disease).

### TSLP Induces an AD-Like Disease Due to Loss of Notch Signaling in the Skin

**Figure 2:**
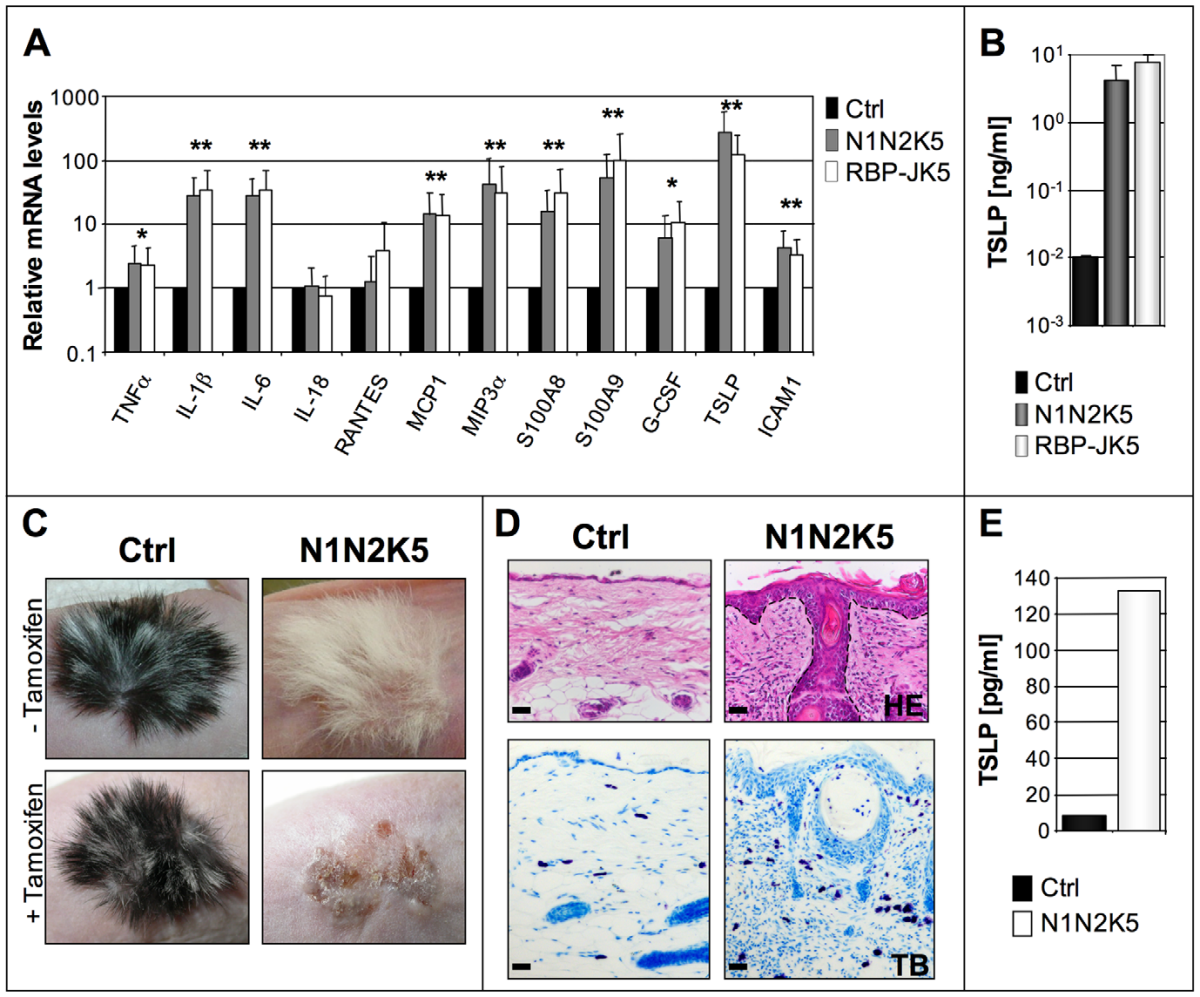
Notch signaling deficient epidermis massively produces TSLP. (**A**) qRT-PCR analysis of inflammatory cytokines on scraped epidermis of control (Ctrl, n = 3), N1N2K5 (n = 3) and RBP-JK5 (n = 3) mice (* p<0.01; ** p<0.001) showing relative increased expression of a wide panel of cytokines in mutant mice. TSLP shows the highest relative increase (125 fold) in mRNA among the tested cytokines. Three individual experiments were performed. (**B**) Serum TSLP levels in control (Ctrl, n = 4), N1N2K5 (n = 4) and RBP-JK5 (n = 4) mice revealing a 400 fold increase of this cytokine in mutant mice. The experiment was performed in triplicates. (**C-D**) Control (Ctrl) and N1N2K5 new born skin was grafted onto Athymic *nu/nu* mice and allowed to grow for 2 months (-Tamoxifen, n = 3). After induction of Cre-mediated recombination (+Tamoxifen, n = 3), the graft develops a similar phenotype to N1N2K5 mice. H/E and Toluidin blue staining shows acanthosis, hyperkeratosis, spongiosis, epidermoid cysts and massive infiltration of mast cells in the dermis of the N1N2K5 derived graft. Three individual experiments were performed. (**E**) TSLP serum levels of athymic nu/nu mice after grafting the skin of control (Ctrl) or N1N2K5 mice and subsequent gene inactivation. Serum from 3 Ctrl and 3 grafted Athymic *nu/nu* mice were pooled for the analysis. Bars represent the mean of two individual experiments.

Consequently, we investigated more specifically the strong inflammatory response within the Notch deficient epidermis. For this purpose, we selectively isolated RNA from the epidermis of control, N1N2K5 and RBP-JK5 mice and conducted quantitative real-time PCR analysis for a panel of inflammatory cytokines, many of which were significantly increased (Figure 2A). These included TNFα (2.5 fold), IL-1β (28 fold) and IL-6 (27 fold), MCP1 (15 fold), MIP3α (43 fold), S100A8 (15 fold), S100A9 (56 fold), G-CSF (6 fold) and ICAM1 (3.5 fold). However, the cytokine TSLP showed the highest increase in relative amount (up to 125 fold), in both N1N2 and RBP-J deficient epidermis. Other *Keratin 5*-expressing tissues such as the thymic epithelium or bone marrow (BM) cells did not show increased TSLP expression (data not shown). Protein levels of TNFα and IL-1β were similar throughout the serum samples of mice investigated (data not shown). However, TSLP levels increased 400 fold in N1N2K5 and RBP-JK5 mice. They reached on average 4 to 7 ng/ml respectively (Figure 2B), suggesting that TSLP might be causative for the AD-like phenotype, as previously reported in humans and mice [22], [23], [24].

It seemed essential to exclude the possibility that the observed phenotypes may be caused by ectopic expression of the Cre recombinase in an organ other than the skin. Thus, we transplanted the skin of control and N1N2K5 newborn pups onto athymic *nu/nu* mice. Two months post-transplantation, the grafted mice were injected with tamoxifen in order to simultaneously inactivate both Notch receptors within the skin transplants. Histological analysis of the skin grafts derived from N1N2K5 mice, but not from control mice, showed a thickened and hyperkeratinized epidermis, epidermoid cysts and dermal inflammatory infiltrates characterized by the recruitment of mast cells (Figure 2C, D). In addition, sera of nude mice transplanted with N1N2K5 skin showed a 13-fold increase in TSLP protein levels compared to control sera (Figure 2E). These results strongly suggest that the AD-like phenotype is indeed a consequence of the specific loss of Notch signaling in the epidermis.

To investigate whether TSLP expression in the skin is sufficient to cause AD, transgenic mice expressing TSLP under the *Keratin14* promoter (K14-TSLP) were compared to the Notch deficient animals. K14-TSLP mice exhibit a nearly identical skin phenotype to Notch signaling deficient mice, showing hyperproliferation, acanthosis, spongiosis and hyperkeratosis, as well as mast cell infiltration in the dermis, but normal hair follicles (Figure S4). Together, these data indicate that TSLP in this setting is sufficient to cause AD in mice, which is consistent with a previous report [23].

### Notch Receptors Are Downregulated in the Skin of AD Patients

**Figure 3:**
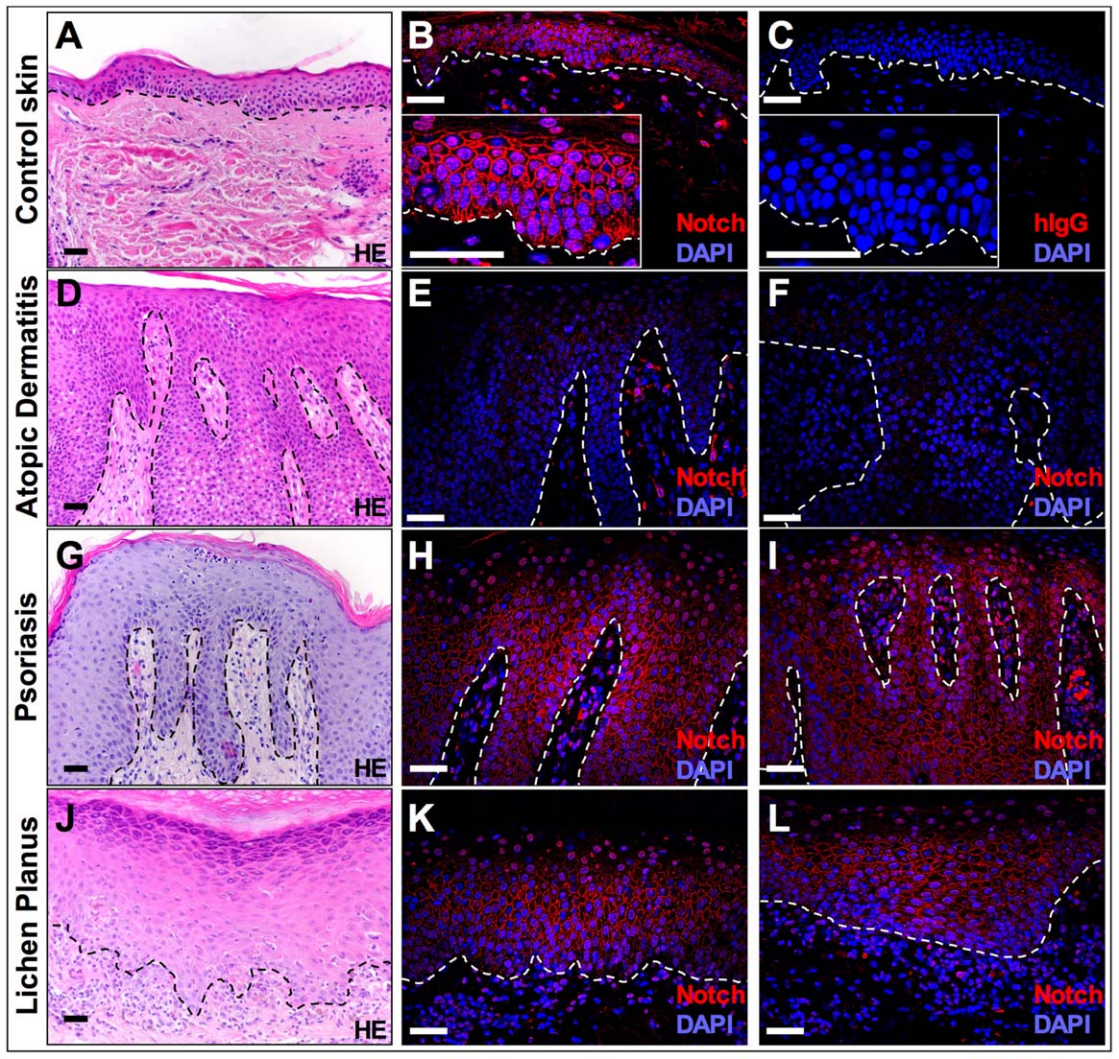
Notch receptor expression is down regulated in skin samples of AD patients. H/E staining of representative skin sections derived from (**A**) human control skin (n = 9) and lesional sites from the following human skin disorders: (**D**) atopic dermatitis (n = 9), (**G**) psoriasis (n = 4), and (**J**) lichen planus (n = 4). (**B**, **E**, **F**, **H**, **I**, **K** and **L**) show panNotch staining using a DL4-IgG fusion protein while (**C**) shows control staining with a hIgG isotype control antibody. Nuclei are counterstained with DAPI. (**E**-**F)** shows down regulation of Notch receptor expression in skin sections from two different AD patients, while (**H**-**I**) reveals the presence of Notch expression on sections from two psoriasis patients and (**K**-**L**) from two patients suffering from lichen planus. [Scale bars: 50 µm].

Since loss of Notch signaling and TSLP expression are linked in mouse skin, we assessed a possible role for Notch in the etiology of AD in humans. Therefore, we analyzed the presence of Notch receptors with a tagged ligand (DL4-FC) [17] that recognizes presumably all four Notch paralogs in affected areas of biopsy samples from AD patients (n = 9) compared to control patients (n = 9). Notch receptor expression is confined to the suprabasal cell layer of unaffected skin samples (Figure 3A-C). In contrast, Notch receptor protein expression was greatly reduced or even no longer detectable in lesional skin in 7 out of 9 AD patients (Figure 3D-F). As Notch has been shown to induce early differentiation events and has been linked to cell cycle withdrawal, it is possible that diminished Notch receptor expression is common to many hyperproliferative skin disorders rather then specific to AD. We therefore analyzed biopsy samples from psoriasis (n = 4) and lichen planus patients (n = 4). Patient tissue samples from both of these hyperproliferative skin disorders showed a strong presence of Notch receptors (Figure 3G-L) indicating that Notch receptor regulation and signaling is closely linked to AD and not to the other skin disorders tested.

### Epidermal-Specific Loss of Notch Signaling Provokes a Microenvironment-Induced Myeloproliferative Disorder (MPD)

**Figure 4:**
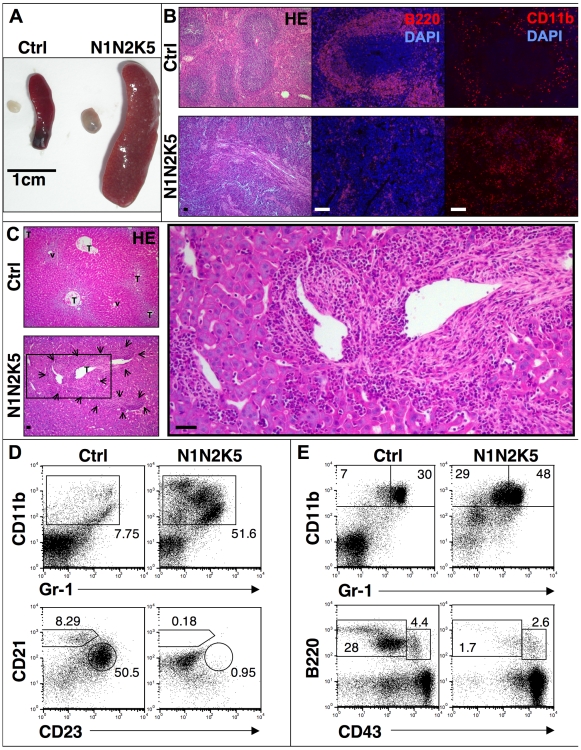
N1N2K5 mice develop a myeloproliferative disorder (MPD). (**A**) Representative images of spleen and lymph node of control (Ctrl) and N1N2K5 mice showing splenomegaly and lymphadenopathy (n = 12, three individual experiments). (**B**) HE staining, B220 and CD11b immunofluorescence on spleen sections showing a loss of normal splenic architecture with fibrosis, loss of follicular structures (B220^+^ B cells) and increase in CD11b^+^ myeloid cells in N1N2K5 mice (n = 12, three individual experiments). (**C**) Representative HE staining on liver sections from control and N1N2K5 mice. The liver structure with terminal hepatic venules (v) and portal tracts (T) is changed due to periportal invasion of inflammatory cells and fibrotic reactions in Notch mutant mice (arrows and insert, n = 8, two individual experiments). (**D**) Representative flow cytometric analysis of splenic myeloid and B cells showing a massive increase in myeloid cells (CD11b^+^Gr-1^int^) and loss of follicular (B220^+^CD23^+^CD21^int^) and marginal zone B cells (B220^+^CD23^lo/-^CD21^+^) in N1N2K5 mice. (**E**) Representative cytometric analysis of bone marrow myeloid and B cells showing an increase in myeloid (CD11b^+^Gr-1^int^) and a block of B cell development at the pre-pro B stage (B220^+^CD43^+^). Numbers indicate the percentage of cells in each gate. Results are representative of n = 12 per sample group of three individual experiments. [Scale bars: 50 µm].

Despite the fact that decreased Notch receptor expression is found in both AD and squamous cell carcinoma (SCC) patients [25], AD patients do not seem to have an increased risk of developing skin malignancies [26]. In contrast, they have an increased probability of developing hematopoietic malignancies and this risk seems to correlate with the severity of AD [27], [28], [29]. Interestingly, autopsy of N1N2K5 and RBP-JK5 mice revealed the development of an apparent myeloproliferative disorder (MPD), which may account for their early death. N1N2K5 and RBP-JK5 mice developed splenomegaly, as well as lymphadenopathy (Figure 4A). This was characterized by a 2-3 fold increase in absolute cell numbers of splenocytes and lymph node cells (Figure S5). Histological and immunohistochemical analysis of the spleen revealed loss of the red and white pulp, splenic fibrosis, loss of B cell follicles and an increase in myeloid cells (Figure 4B), as well as periportal inflammatory infiltrations in the liver (Figure 4C). Flow cytometric (FC) analysis of splenocytes derived from control and N1N2K5 mice confirmed the immunohistochemistry and showed a dramatic increase, in relative percentages and absolute numbers, of mature and immature granulocytes (CD11b^+^Gr-1^+^ and CD11b^+^Gr-1^lo/int^), and a nearly complete loss of splenic follicular (B220^+^CD23^+^CD21^int^) and marginal zone (B220^+^CD23^lo/int^CD21^+^) B cells (Figure 4D, Figure S5). Further analysis of the BM showed decreased absolute cellularity, revealed an accumulation of immature granulocytes (CD11b^+^Gr-1^lo/int^) and a block in B cell development at the (B220^+^CD43^+^) pre-pro B cell stage (Figure 4E). Cell cycle analysis also showed increased cycling activity of the granulocytic cell population in the spleen of N1N2K5 and RBP-JK5 mice (data not shown). Taken together, these data strongly suggest that epidermal-specific loss of Notch signaling results in a MPD.

As MPD may occur due to neoplastic alterations intrinsic to hematopoietic cells, we ensured that the phenotype observed was not due to aberrant Cre-recombinase activity in hematopoietic progenitors. To exclude this possibility we generated BM chimeras by transplanting CD45.1^+^ wild-type BM into CD45.2^+^ control, N1N2K5 or RBP-JK5 mice. The reconstitution efficiency for control, N1N2K5 and RBP-JK5 chimeras was >80% (data not shown). The hematopoietic system of N1N2K5 (data not shown) and RBP-JK5, but not control, BM chimeras (which are reconstituted from WT cells) developed an identical hematopoietic phenotype to the *Notch* mutant mice (Figure S6A). Reciprocal transplantation of CD45.2^+^ BM cells from diseased RBP-JK5 mice into CD45.1^+^ wild-type recipient mice (reconstitution efficiency >80%) led to normal hematopoietic development as expected (Figure S6B). These results demonstrate that the MPD-like phenotype observed in N1N2K5 and RBP-JK5 mice is caused by systemic cell non-autonomous effects due to loss of Notch signaling in the skin. This result is similar to the cell non-autonomous B-LPD that was observed in mice in which Notch signaling was abrogated during embryogenesis [13].

### Excessive TSLP-Mediated Signaling in Skin Specific Notch Mutant Mice Is Causative for Both the AD- and MPD-Like Phenotypes

**Figure 5:**
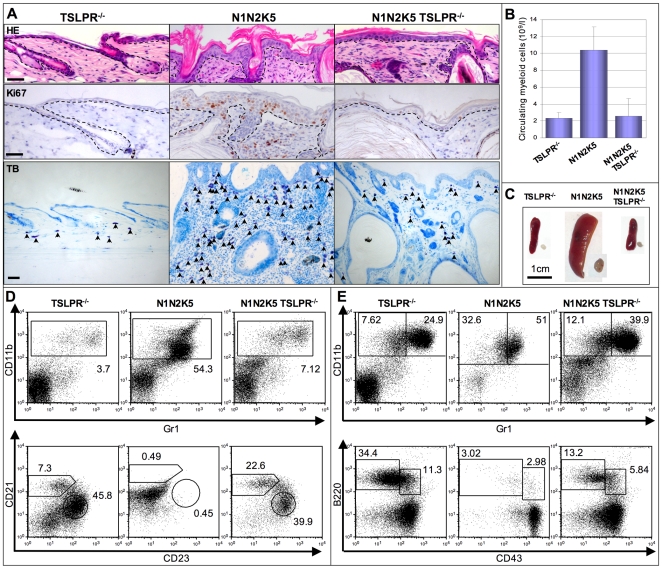
TSLP is causative of both AD and MPD in N1N2K5 mice. TSLPR^−/−^ mice were indistinguishable from wild type mice and therefore only the results of TSLPR^−/−^ mice are shown. (**A**) Representative skin sections of TSLPR^−/−^, N1N2K5 and N1N2K5 TSLPR^−/−^ mice stained for HE (upper panels), Ki67 (middle panels) and Toluidin blue (lower panels). Notch mutant mice lacking TSLPR have a markedly less proliferative epidermis, do not develop spongiosis and have large a reduction in dermal inflammatory cells (arrows, n = 6 per sample group, three individual experiments). (**B**) Myeloid cell counts in peripheral blood, and (**C**) spleen and lymph node (LN) macroscopy from TSLPR^−/−^, N1N2K5 and N1N2K5TSLPR^−/−^ mice showing a rescue of the MPD phenotype. The bar diagrams represent mean values ± SD (n = 3 for each genotype of mice, three individual experiments). Representative flow cytometric analysis of myeloid and B cells of the spleen (**D**) and bone marrow (**E**) from TSLPR^−/−^, N1N2K5 and N1N2K5 TSLPR^−/−^ mice stained for CD11b and Gr1, CD21 and CD23 (gated on B220^+^ splenic B cells), or B220 and CD43 (n = 6 per sample group, three individual experiments). [Scale bars: 50 µm].

We assessed whether excessive TSLP-mediated signaling is indeed responsible for the development of both the AD-like and MPD phenotypes. For this purpose TSLPR^−/−^ mice [30] were intercrossed with N1N2K5 mice in order to generate triple mutants (N1N2K5 TSLPR^−/−^). As TSLPR^−/−^ mice were identical to wild type mice in our analyses, we used the former as controls. Additional loss of TSLPR led to a markedly less proliferative epidermis, which was also devoid of spongiosis and displayed a substantial decrease in inflammatory dermal infiltrates compared to N1N2K5 animals (Figure 5A). Moreover, myeloid cell counts in peripheral blood from N1N2K5 TSLPR^−/−^ mice were comparable to controls, while they were increased 5-fold in N1N2K5 mice (Figure 5B). N1N2K5 TSLPR^−/−^ mice had normal sized spleens and lymph nodes (Figure 5C). FC analysis of splenocytes and BM cells of the three different genetic groups of mice showed that the absence of TSLPR in N1N2K5 mice was sufficient to restore normal hematopoiesis. The spleen and BM of N1N2K5 TSLPR^−/−^ mice revealed the presence of normal granulocyte numbers and no block in B cell development (Figure 5D, E). These findings clearly reveal that both AD and the MPD are mediated by excessive TSLP levels.

### TSLP-Induced MPD in Epidermal-Specific Notch Mutant Mice Is Mediated by G-CSF

**Figure 6:**
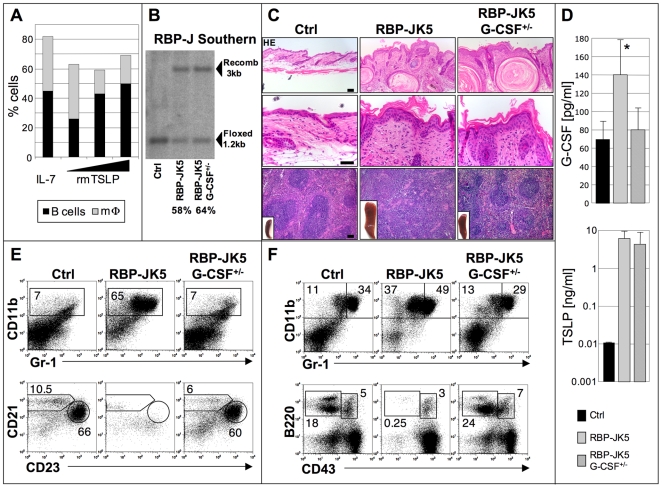
G-CSF is responsible for the cell non-autonomous development of the TSLP-induced MPD. (**A**) EPLM cultures on ST-2 cells in presence of IL-7, or increasing concentrations of rmTSLP showing an increase in B cell numbers, but no increase of myeloid cells. The experiment was performed twice, and each cytokine concentration was analyzed as triplicates. (**B**) Southern blot analysis of genomic DNA from scraped epidermis from control (Ctrl), RBP-JK5, and RBP-JK5G-CSF^+/−^ mice showing the floxed and the recombined (Recomb) alleles of the RBP-J gene. Recombination efficiency is similar for the two mutant mouse strains. Representative blot for n = 3 mice of each genotype, three individual experiments. (**C**) HE staining on skin (two upper panels) and spleen (bottom panels) sections from control (Ctrl), RBP-JK5 and RBP-JK5/G-CSF^+/−^ mice. Macroscopy of the spleens in inserts (n = 5 mice per sample group from two individual experiments). (**D**) Serum G-CSF and TSLP levels in control (Ctrl), RBP-JK5 and RBP-JK5/G-CSF^+/−^ mice (* = p value<0.02). The bar diagrams represent mean values ± SD (n = 5 per sample group of mice from two individual experiments). Representative flow cytometric analysis of myeloid and B cells of the spleen (**E**) and the bone marrow (**F**) from control (Ctrl), RBP-JK5 and RBP-JK5/G-CSF^+/−^ mice stained for CD11b and Gr1, or CD21 and CD23 (gated on B220^+^ splenic B cells), or B220 and CD43 within the BM compartment (n = 5 per sample group from two individual experiments). [Scale bars: 50 µm].

As TSLP is mostly known to influence and/or promote B cell development [31], [32], [33], it is not clear how it can induce a MPD. To determine whether TSLP functions in a direct manner to cause the MPD in N1N2K5 mice, we investigated the influence of TSLP on hematopoietic differentiation. WT early BM progenitors with lymphoid and myeloid potential (EPLM: CD117^+^B220^int/+^CD93^+^CD19^-^CD3^-^NK1.1^-^) [34] were cultured on stromal ST-2 cells in the presence of IL-7, an essential cytokine to promote B cell development, or with increasing concentrations of recombinant murine TSLP. BM progenitors cultured with TSLP (0.5-50 ng/ml) showed a developmental skew towards the B cell lineage, implying that TSLP can substitute for IL-7 and thus favors B cell development, which is in agreement with previous reports [31], [32], [33]. Even high concentrations of TSLP in the culture medium did not lead to an expansion of myeloid cells (Figure 6A) suggesting that TSLP cannot enhance proliferation or differentiation of immature progenitors into the myeloid lineage in a cell autonomous manner.

The increase of immature granulocytes in the spleen and BM (Figure 4D-E) of N1N2K5 or RBP-JK5 mice phenotypically resembles mice that have been injected with G-CSF [35]. Moreover, the mutant mice developed osteopenia with loss of the endosteal lining (Figure S7). A similar phenotype is observed in patients receiving G-CSF therapy, who often suffer from osteoporosis as a side effect [36]. This prompted us to investigate whether G-CSF might be responsible for the myeloid hyperproliferation. We first assessed G-CSF levels in sera of control, N1N2K5 and RBP-JK5 mice. Both *Notch* mutant mice showed a significant increase in G-CSF serum levels compared to control animals (Figure 6D and data not shown). In contrast, GM-CSF protein levels, another myeloid-promoting cytokine, were unchanged (data not shown). We therefore hypothesized that decreasing G-CSF concentration in *Notch* mutant mice would rescue the hematopoietic phenotype. For this purpose we intercrossed RBP-JK5 mice with *G-CSF^-/-^* mice to generate RBP-JK5/G-CSF^+/−^ mice. RBP-JK5/G-CSF^+/−^ had serum levels of G-CSF similar to control mice (Figure 6D). Strikingly, the size as well as the histology of spleens derived from RBP-JK5/G-CSF^+/−^ mice was comparable to control animals, while RBP-JK5 mice showed clear splenomegaly with loss of the white and red pulp (Figure 6C). Furthermore, FC analysis of RBP-JK5/G-CSF^+/−^ mice showed a complete rescue of the hematopoietic phenotype characterized by a normal B cell and myeloid pattern in the BM and spleen (Figure 6E and 6F). Importantly, the deletion efficiency of the RBP-J gene in RBP-JK5 and RBP-JK5/G-CSF^+/−^ mice was comparable, suggesting that the rescue of the hematopoietic phenotype was not simply the consequence of unequal Cre-mediated recombination efficiency (Figure 6B). Moreover, analysis of bone sections revealed an intact endosteal lining (Figure S7), suggesting that G-CSF may modulate bone homeostasis through osteoblasts. Histological analysis of the skin of control, RBP-JK5 and RBP-JK5/G-CSF^+/−^ mice showed the development of an AD-like disease in both mutant mice, implying that reduced G-CSF serum levels did not ameliorate the skin phenotype (Figure 6C), which is expected as TSLP serum levels are still elevated in the RBPJ-K5/G-CSF^+/−^ mice (Figure 6D).

However, the median survival of RBP-JK5/G-CSF^+/−^ compared to RBP-JK5 mice doubled (105 versus 52 days, n = 14), suggesting that the primary cause of death of Notch mutant mice is the G-CSF-induced MPD and not the skin disorder.

## Discussion

N1N2K5 and RBP-JK5, (but not N1K5 or N2K5) mice show clinical, histological and cellular features that are typical of human AD. AD is the most common childhood skin disorder [37], disappearing in most cases with age. However, in some patients the disease persists with severe clinical consequences. The cause of AD is currently unknown, but its pathology has been linked to the production of inflammatory cytokines including TNF-α, IL-1β, IL-4, IL-5 and others [38], [39]. Analysis of *Notch* mutant skin revealed an increased expression of multiple inflammatory cytokines and dermal infiltration of Th2 cells. However, secretion of the Th17 cytokines Il-21 and IL-22 that have previously been linked to epidermal hyperplasia [40], [41] was not affected. IL-17 expression is increased in the skin of acute AD patients [42], [43], while it seems to be decreased in chronic AD patients [44], [45]. *In vitro*, stimulation of mouse CD4^+^ T cells under Th2 conditions has been shown to abolish IL-17 and IL-22 production [40]. Thus, it is possible that the onset of a Th2 response in the *Notch* mutant skin may block the Th17 immune response.

TSLP was the cytokine found to be most dramatically increased in both N1N2K5 and RBP-JK5 mutant mice. TSLP is an IL-7-like cytokine and is able to support B cell differentiation and T cell proliferation [46], [47], with an ability to act on both CD4^+^ and CD8^+^ T cells [48], [49] as well as on DC [50]. Subsequent studies showed that TSLP expression is highly upregulated in keratinocytes of AD patients [22] and it is also involved in allergic airway inflammation [51], [52]. It is associated with the activation of DC in the skin causing the production of Th2 cell attracting chemokines [53], and activation of naïve CD4 T cells which subsequently produce inflammatory cytokines [22]. In this context, it is interesting to note that our transplantation experiments of N1N2K5 skin onto Athymic *nu/nu* mice also led to an accumulation of dermal mast cells and the development of AD-like features, despite the absence of functional T cells. In addition, the small transplant was sufficient to markedly increase the TSLP serum levels of the recipient nude mice. These findings are in agreement with reports showing that mice overexpressing TSLP, but lacking T or B cells (TCRβ^−/−^, RAG^−/−^), also develop allergic disorders at epithelial surfaces [23], [51], [54]. Activated mast cells, which can also produce high levels of Th2 cytokines in response to TSLP, have been identified as possible cellular mediators of allergic disorders [54].

Postnatal inactivation of Notch signaling in murine skin leads to hyperkeratinization of the epidermis, formation of epidermoid cysts in the dermis, and loss of subcutaneous fat. Hyperkeratosis could be the result of deregulated Notch-dependent control of terminal differentiation [5]. Moreover, both loss and gain of Notch function cause hyperkeratosis as a consequence of hair cycle disturbance [7], [14], [55]. Formation of epidermal cysts is the consequence of loss of Notch signaling within hair follicles, which results in the activation of an epidermal differentiation program in the outer root sheet [7]. Abnormalities in fat deposition were only observed in the dermis, a phenotype that was previously reported in mice with skin-specific embryonic mosaic inactivation of Notch signaling [7]. Knowing that canonical Notch signaling is dispensable for adipocyte specification [56], it is likely that loss of the subcutaneous fat is a cell non-autonomous consequence of both epidermoid cyst formation and inflammatory infiltrates.

Loss of Notch signaling in the developing epidermis of the embryo or neonates results in a loss of epithelial barrier function [10], which was suggested to lead to the induction of TSLP expression [13]. In this scenario TSLP expression was suggested to be the indirect consequence of skin barrier defects. A number of susceptibility genes have been identified in AD patients, many of which are expressed in the epithelium (including SPINK5 and Filaggrin) supporting the concept that reduced barrier function, combined with a massive inflammatory response is at the origin of AD [57], [58].

However, additional cell intrinsic mechanisms within skin epithelial cells have recently been shown to contribute to TSLP expression in the absence of barrier defects or microbial products [59]. In this context it is interesting to note that all skin specific expression markers (including LEKTI and Desmoglein – Figure S8) are expressed at normal levels in adult Notch mutant mice suggesting that the skin barrier is unharmed. Moreover, minor barrier function defects, if present at all, are not the cause of death. The Notch mutant mice rather succumb to MPD by high G-CSF levels since lowering G-CSF concentrations in Notch mutant mice is sufficient to drastically prolong their survival. Thus, other cell intrinsic mechanisms must account and/or contribute to TSLP expression in adult Notch mutant mice. Additional signaling cascades, including retinoid X receptor (RXR) signaling and vitamin D3 receptor (VDR) signaling, have also been linked to TSLP-induced AD [24], [60]. Postnatal skin-specific inactivation of both RXRα and RXRβ (RXRs) leads to the development of an AD-like disease characterized by partial hair loss, Th2 inflammation, eosinophilia, elevated TSLP epidermal secretion, and high TSLP serum levels [24]. Although all of these phenotypic hallmarks are shared between RXRs and *Notch* mutant mice, they also exhibit certain differences; for example disease progression of the RXRs mutant mice appears to be milder, it affects primarily ear skin and later to a lesser extent dorsal skin. Furthermore, RXRs mutant mice do not develop a fatal MPD or osteopenia, presumably because of the lower (10-15 x) TSLP serum levels. Consequently RXRs mutant mice live considerably longer compared to Notch mutant mice. So far we could not link the loss of Notch signaling in the epidermis to changes in retinoic acid or vitamin D signaling (data not shown).

We show that human AD patients have reduced Notch receptor levels on the cell surface of the suprabasal epithelium and they are known to have an increased risk to develop hematological malignancies [27], [28], [29]. Thus, it is interesting to note that adult Notch mutant mice develop a severe MPD. Hematological malignancies are generally considered to be the consequence of neoplastic lesions within hematopoietic cells [61], [62], [63]. However, our BM transplantation experiments combined with the genetic rescue studies using TSLPR^−/−^ and G-CSF^+/−^ mice demonstrate that the MPD is the result of a cell non-autonomous process caused by TSLP-induced G-CSF secretion. The G-CSF producing cell-type is currently unknown. Therefore, our results confirm and strongly support a new concept in which changes within the microenvironment can lead to the induction of MPD or other hematological malignancies [13], [64], [65].

TSLP being responsible for the development of the MPD was unexpected. Most reports involve TSLP in B cell homeostasis and/or B cell development, together with its implication in the development of allergic diseases. TSLP was originally described as a cytokine, which leads to increased B cell differentiation when added to BM B cell progenitors [31], [32], [46]. The main TSLP responsive B cell target seems to be a late pro B cell (B220^+^CD43^+^BP1^+^CD24^+^) [66], which in response to TSLP shows increased cell cycle progression [33]. Fetal B cell progenitors seem to be even more responsive to TSLP compared to adult B cell progenitors [66]. This may also explain why high TSLP levels in fetal or neonatal Notch mutant mice cause a B-LPD [13], while the effect on B cells in adult mice is less dramatic. Interestingly, loss of the IL-7Rα or the TSLPR chain, both of which compose the heterodimeric TSLP receptor, rescues both the B-LPD and to a large extent the MPD in mice in which the RBP-J or the presenilin genes were inactivated during embryogenesis (Figure S9). Although most studies indicate that TSLP influences the development and homeostasis of B cells, it has also been reported to have growth promoting functions for myeloid cells. In particular, transgenic overexpression of TSLP using the actin promoter [67] or the myeloid-specific Fes promoter (unpublished observations [33]) resulted in mice with myeloid hyperplasia characterized by increased numbers of myeloid cells in the spleen, which was accompanied by a block in early BM B cell development. Although, both N1N2K5 and RBP-JK5 mice develop a MPD, this was not observed in K14-TSLP tg mice or in the grafted athymic nu/nu mice which had 10-50x lower TSLP serum levels, suggesting that TSLP can only induce a MPD, when present at high concentrations. Thus, our studies provide the first genetic proof that high TSLP concentrations are causative for the development of a cell non-autonomous MPD. The nature of the hematological disorder is dependent on the age of the mice (embryonic versus adult) while the development of the MPD appears to depend on the TSLP concentration. TSLP serum levels have not been studied in AD patients. As they are known to have an increased risk of developing hematopoietic malignancies correlating with the severity of their dermatitis [27], [28], [29], we analyzed serum from AD patients and found 2 out of 4 patients had increased TSLP levels (50 and 57 pg/ml compared to undetectable levels in all controls). Thus it will be interesting in future studies to correlate patient TSLP serum levels with the risk of developing hematological malignancies.

**Figure 7:**
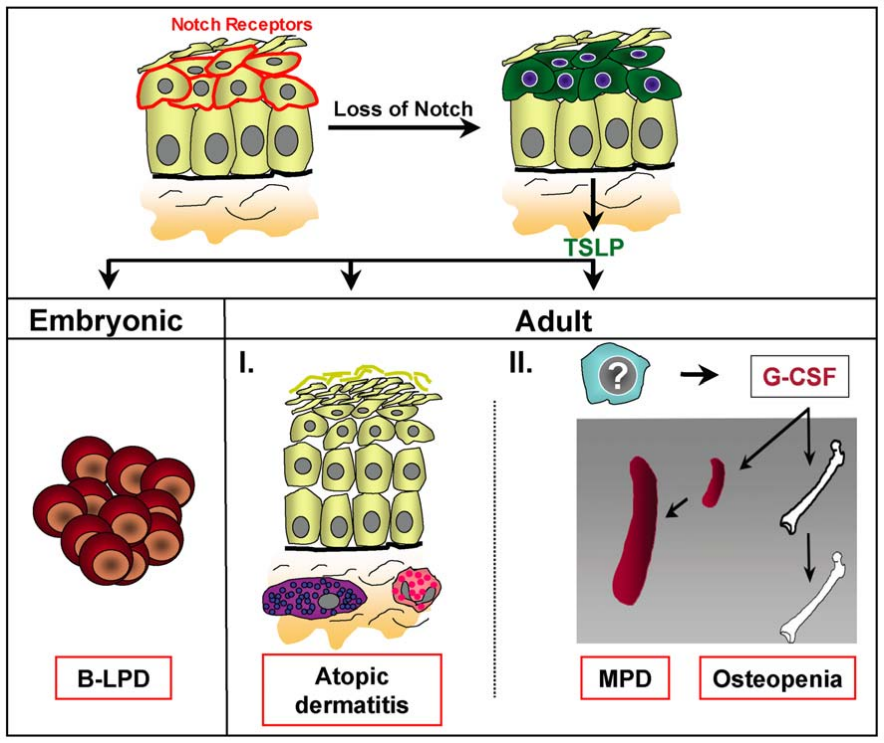
Model for the role of Notch signaling in adult skin and how its loss results in the development of AD, MPD and oteopenia. Notch receptors are expressed in the suprabasal cell layer of the skin. Skin specific loss of Notch signaling leads to pronounced secretion of TSLP by epithelial cells. High TSLP serum levels in the embryo or neonates cause a cell non-autonomous B-LPD. In contrast, the presence of TSLP in adult mice results in the recruitment of mast cells and eosinophiles within the dermis of Notch mutant mice, thereby contributing to massive inflammation and the development of an AD-like disease. At very high TSLP serum levels, G-CSF is produced by a currently unknown cell type, causing the cell non-autonomous development of MPD and osteopenia.

In conclusion, our studies reveal novel aspects of Notch signaling in adult versus embryonic skin; it is essential to control local and systemic inflammatory responses. Loss of Notch signaling in the embryonic skin leads to a cell non-autonomous B-cell lymphoproliferative disease as previously reported [13]. However, Notch inactivation in the adult skin does not cause B-LPD; instead it causes an AD-like disease accompanied by a cell non-autonomous G-CSF induced MPD and osteopenia. Our genetic studies reveal that TSLP receptor mediated signaling is causative for the different hematological disorders (Figure 7).

## Materials and Methods

### Mice

*Notch1^lox/lox^* [68], *Notch2^lox/lox^*, *Notch1^lox/lox^ Notch2^lox/lox^* [17] and *RBP-J^lox/lox^* [18] mice were crossed with *K5Cre^ERT^* [19] transgenic mice. TSLPR^−/−^ [30] mice were crossed to N1N2K5 mice to generate N1N2K5 TSLPR^−/−^ mice. *G-CSF^-/-^* mice [69] were crossed to *RBP-JK5* mice to generate *RBP-JK5/G-CSF^+/−^* mice. For gene inactivation 8-day-old control and floxed Notch mutant mice were injected with 1mg/20g body weight of tamoxifen (Sigma-Aldrich, Switzerland) for 5 consecutive days. *K14-TSLP* transgenic, IL-7Rα^−/−^ [70] and Msx2Cre mice were previously described [7], [71]. Athymic (Swiss *nu/nu*, inbred) mice used for the transplantation experiments were purchased from Iffa Credo-Charles River (France).

### Ethics Statement

All animal work was conducted according to Swiss national guidelines. All mice were kept in the animal facility under EPFL animal care regulations. They were housed in individual cages at 23±1°C with a 12-h light /dark cycle. All animals were supplied with food and water ad libitum. This study has been reviewed and approved by the Service Vétérinaire Cantonal of Etat de Vaud.

### Flow Cytometry and Cell Sorting

Single-cell suspensions of BM were prepared and stained following standard protocols for fluorescence-activated cell-sorter scanner (FACS) analysis using the following monoclonal antibody conjugates: CD43-FITC (clone S7, BD Pharmingen), Gr-1-PE-Cy7 (clone RB5-8C5, ebiosciences); B220 (RA3.6B2)–Alexa Fluor 647; CD11b (M1/70)-Alexa Fluor 647 and CD45.2 (104)–PE. All antibodies were purified from hybridoma supernatants and conjugated in our laboratory according to standard protocols. Alexa Fluor 647 conjugates were prepared using the appropriate Alexa Fluor protein labeling kits (Invitrogen). PE conjugates were prepared using kits purchased from Prozyme. Single-cell suspensions were stained with the respective antibodies and analyzed using a FACSCalibur, FACSCanto (Becton Dickinson) or CyAn flow cytometer (Dako). Dead cells and debris were eliminated by appropriate gating on forward and side scatter. The data were analyzed using FlowJo (TreeStar, Inc.) software. EPLMs were stained and sorted on a FACSAria flow cytometer (Becton Dickinson) as previously described (Balciunaite et al., 2005). Sorted EPLMs were culture on irradiated (3000 rads) ST2 stromal cells in the presence of recombinant murine TSLP (555-TS-010, R&S Systems).

### Quantitative RT-PCR

Keratinocytes were scraped from frozen skin of control or N1N2K5 and RBP-JK5 mice and total RNA was isolated using TRIZOL reagent (Invitrogen). RNA was quantified using a ND-100 NanoDrop spectrophotometer (NanoDrop Technologies). 1 µg of total RNA was reverse-transcribed using the Quantitect reverse transcription kit (Qiagen). 18S was used to control for equal cDNA inputs. Real-time PCR was conducted with a LightCycler system (Roche Diagnostics). Reactions were performed using primers and template mixed with the LightCycle DNA master SYBR Green kit and run for 45 cycles. Specificity of the reactions was determined by subsequent melting curve analysis. LightCycler analysis software was used for quantifications, and background fluorescence was removed using the noise band. The number of cycles needed to reach the crossing point for each sample was used to calculate the amount of each product using the 2^-ΔΔCP^ method. Relative levels of expression were normalized to 18S or HPRT expression.

### PanNotch Staining

Paraffin embedded skin samples were sectioned at 4 µm. The sections were dewaxed and antigen retrieval was performed in trisodium citrate solution at 95°C for 20′. Sections were blocked in 1% BSA in TBS-Tween for 45 minutes. 20 µg/ml of Delta4-IgG fusion protein [17] or of human IgG (Caltag #12400C) was then added and the slides were hybridized over night at 4°C. The sections were then stained with an Alexa Fluor® 568 conjugated goat anti-human IgG (H+L) diluted at 1 in 500 (Invitrogen/Molecular Probes #A-21090) for 1h at room temperature. Nuclei were stained with DAPI (D-9542, Sigma). The sections were mounted with DABCO (D-2522, Sigma). Photos were taken with an Axioplan microscope with an Apotome (Zeiss).

Additional experimental procedures can be found online as supporting information (Data S1).

## Supporting Information

Data S1 Supplementary Experimental Procedures
(0.04 MB DOC)

Figure S1 Loss of both Notch1 and Notch2, or RBP-J, but not Notch1 or Notch2 alone, leads to the development of a severe skin phenotype. Representative HE staining, Keratin14 (K14), Keratin1 (K1), Loricrin (Lori) and Ki67 immunohistochemistry and Toluidine Blue (TB) staining on dorsal skin sections from control (Ctrl), N1K5, N2K5, N1N2K5 and RBP-JK5 mice (all within 30-40 days of tamoxifen injection) (n = 8, from 4 individual experiments). Mice lacking Notch2 in the epidermis are indistinguishable from controls, whereas Notch1 deficient mice show K14 expression throughout the epidermis (whereas it is confined to the basal layer in controls), have a hyperproliferative epidermis and a mild increase in toluidine blue positive mast cells. Loss of Notch signaling in N1N2K5 or RBP-JK5 mice leads to an even more hyperproliferative phenotype with Ki67 positive cells throughout the entire epidermis, which is also K14 positive. However, K1 and Loricrin are still expressed in the upper layers of the epidermis. Loss of Notch signaling also leads to a massive dermal infiltration of mast cells [Scale bars: 50 µm].
(4.69 MB TIF)

Figure S2 Analysis of DCs and other bone marrow derived cells in the skin. (A). Histograms show the staining of CD45 in total dermal cell suspensions from control (Ctrl) or N1N2K5 mice. The percentage of CD45+ cells is indicated. The bar diagram indicates the percentage of T cells (CD3+ cells), B cells (CD19+ cells), dendritic cells (DCs, CD11b+ CD11c+ cells), neutrophils (Ly6G+ cells) and other myeloid cells (CD11b+ CD11c-) within the CD45+ gate in control (Ctrl) or N1N2K5 dermis. Dot plots show analysis of DCs (gated as CD45+ CD11c+) populations in the dermis. CD11b versus CD103 staining displays an increase of dermal Langerin+ DCs (CD11b+ CD103+) in N1N2K5 mice (41.2%) compared to control (Ctrl) mice (21.3%). CD11b versus Ly6C staining shows an increase of inflammatory DCs (CD11b+ LyC+) in N1N2K5 dermis (53.5%) compared to Ctrl dermis (7.5%). Plasmacytoid DCs (CD11b- CD11c+ PDCA1+) were not detected either in control (Ctrl) or N1N2K5 dermis (data not shown). Prior to flow cytometric analysis the CD45 population as well as the populations of DCs were enriched using magnetic MACS cell separation. (B) Histograms show CD45 staining in total epidermal cell suspensions from control (Ctrl) and N1N2K5 mice. The percentage of CD45+ cells is indicated. The bar diagram indicates the relative percentage of murine dendritic epidermal T cells (DETCs, CD3+ Vγ3+), Langerhans cells (LCs, CD11b+ CD11c+), and CD11b+ CD11c- cells within the CD45+ gate in control and N1N2K5 epidermis. Flow cytometric analysis was performed after magnetic MACS cell enrichment. Cells analyzed were a pooled sample size of n = 8 for control or n = 8 for N1N2K5 from two individual experiments.
(0.83 MB TIF)

Figure S3 Quantitative RT-PCR on dermis-derived RNA for the T helper specific cytokines IFNγ, IL-12, IL-13, IL-17a, IL-21, IL-22 from Ctrl and N1N2K5 mice. Two to four individual experiments were performed (n = 3 per sample group); each experiment was run in triplicates (* p<0.01).
(0.19 MB TIF)

Figure S4 Skin-specific transgenic TSLP expression is sufficient to induce a similar AD phenotype as the Notch signaling deficient mice. Representative HE staining, Ki67 immunohistochemistry and Toluidine Blue (TB) staining on skin sections from control (Ctrl, n = 4), N1N2K5 (n = 8) and K14-TSLP mice (n = 4). The results shown are representative of 2 individual experiments. Both N1N2K5 and K14-TSLP mice show acanthosis, hyperkeratosis as well as dermal infiltration by mast cells [Scale bars: 50 µm].
(8.68 MB TIF)

Figure S5 Loss of TSLP signaling or reduction of GCSF levels rescues the MPD. (A) Absolute cell numbers in the spleen of control, N1N2K5, N1N2K5 TSLPR−/−, RPB-JK5 and RBP-JK5/GCSF+/− mice. (B) Splenic absolute cell numbers of immature (CD11b+Gr.1lo/int) and mature (CD11b+Gr.1lo/int) myeloid populations, as well as (C) marginal zone (B220+CD21+CD23lo) and follicular (B220+CD21intCD23+) B cells. (n = 5 per sample group from two individual experiments).
(0.42 MB TIF)

Figure S6 The MPD in mice lacking Notch signaling in the epidermis is cell non-autonomous. (A) Representative flow cytometric analysis of chimeras (WT CD45.1+ BM cells derived from n = 3 mice were transplanted into 9 lethally irradiated RBP-JK5 mice). WT cells adopt a similar myeloproliferative phenotype in the BM and spleen as in the RBP-JK5 mice. (B) Representative flow cytometric analysis of reverse chimeras (CD45.2+ BM cells derived from 3 sick RBP-JK5 mice were intravenously transplanted into 9 lethally irradiated WT CD45.1+ recipients). RBP-JK5 derived BM cells show normal hematopoiesis in a WT environment. The results in A and B are derived from 3 individual experiments.
(2.40 MB TIF)

Figure S7 Loss of Notch signaling in the epidermis leads to osteopenia and is due to high G-CSF levels. (A) Representative X-ray analysis of femurs from control (Ctrl) and N1N2K5 mice showing a marked decrease in bone density in the mutant bone. (B) Femur length of control (Ctrl) and N1N2K5 mice (* = p value<0.05, n = 10, three individual experiments). (C) Femur cortical thickness of control (Ctrl) and N1N2K5 mice (* =  p value<0.001, n = 10, three individual experiments). (D) Representative HE staining on femoral bone sections from control (Ctrl), N1N2K5, RBP-JK5 and RBP-JK5/G-CSF+/− mice showing a loss of endosteal cells in N1N2K5 and RBP JK5 mice and rescue of this phenotype in the RBP-JK5/G-CSF+/− mice (n = 5 per sample group, two individual experiments). [Scale bars: 50 µm].
(3.62 MB TIF)

Figure S8 Intact stratum corneum in N1N2K5 mice. Down regulation or loss of LEKTI is frequently observed in skin with altered desquamation, impaired keratinization and skin barrier defects. LEKTI deficiency causes abnormal desmosome cleavage in the upper granular layer through degradation of desmoglein 1. This leads to defective stratum corneum adhesion and thus to the loss of barrier function. Positive staining for (A) the multi-domain serine protease inhibitor LEKTI (lympho epithelial kazal-type inhibitor) and (B) Desmoglein indicates the presence of an intact epidermis in both control (Ctrl) and N1N2K5 mice 5 weeks after gene inactivation (n = 3 mice per sample group, two individual experiments). [Scale bars: 50 µm].
(5.28 MB TIF)

Figure S9 TSLP is required for B-LPD occurrence in Notch signaling-deficient animals. TSLP binds to a heterodimeric receptor that shares one of its subunits (IL-7rα) with IL-7 receptor. Inhibiting TSLP effects by removing either TSLPR (TSLPR−/−) or IL-7rα (IL7rα−/−) subunit of TSLP receptor leads to disappearance of B-LPD in the mutant mice. (A) WBC counts of RBP-j-deficient animals lacking IL-7rα (Msx2-Cre/+; RBP-jflox/flox; IL-7rα−/− or RBP-jCKO;IL7rα−/−) are within the normal range at P14. (B) Flow cytometric analysis on peripheral blood shows no signs of B-LPD in RBP-jCKO;IL7rα−/− mice compared to their Msx2-Cre/+; RBP-jflox/flox; IL-7Rα+/− (RBP-jCKO) littermates at P14. Representative results are shown with B220+ B cell percentage (red) in the upper right corner of each dot plot. IL-7rα−/− and wild type littermates are also analyzed as controls. Note that WBC counts and B cell percentage of RBP-jCKO;IL7rα−/− and IL-7rα−/− mice are lower than wild-type (IL-7rα+/−) littermates. This may be due to a simultaneous inhibition of IL-7 reception and reduction in baseline B and T cells especially in adulthood. (C) WBC counts of N1N2CKO animals lacking IL-7rα (Msx2-Cre/+; Notch1flox/flox; Notch2flox/flox; IL-7rα−/− or N1N2CKO;IL7rα−/−) are normal at P14. (D) Similar normalization of WBC counts is seen with PSDCKO animals that have lost the TSLPR arm of TSLP receptor (Msx2-Cre/+; PS1flox/flox; PS2flox/flox; TSLPR−/− or PSDCKO;TSLPR−/−). (E, F) B-LPD prevention leads to increased life span among (E) N1N2CKO;IL7rα−/− and (F) PSDCKO;TSLPR−/− animals compared to their N1N2CKO and PSDCKO littermates respectively (p<0.001, log rank test). In each panel, data are compiled from 4 animals in each group from two individual experiments.
(1.75 MB TIF)
