# Supplementary material for: Atopic Dermatitis-Like Disease and Associated Lethal Myeloproliferative Disorder Arise from Loss of Notch Signaling in the Murine Skin
Source: PLoS One. 2010 Feb 18;5(2):e9258. doi: 10.1371/journal.pone.0009258 (PMC2823782; doi:10.1371/journal.pone.0009258)
Supplement: Data S1 — Supplementary Experimental Procedures (0.04 MB DOC) [file pone.0009258.s001.doc]

**Supplementary experimental procedures**

**Primers used for pro‑inflammatory cytokine analysis by qRT-PCR.**

Primer sequences were previously described [1]. Specific primers were:

G‑CSF up: GCTGCTGCTGTGGCAAAGT; down: AGCCTGACAGTGACCAGG

IL-17a up: ACCGCAATGAAGACCCTGAT; down: CATCTTCTCGACCCTGAAAGTGA

IL-21 up: TGGCTCCTTCAAAAGATGAT; down: AAACTAGTATGTACTCCTGCATTCGTG

IL-22 up: CATGCAGGAGGTGGTACCTT; down: CAGACGCAAGCATTTCTCAG

S100A8 up: GGAATCACCATGCCCTCTACA; down: TGCCACGCCCACCCTTATC

S100A9 up: GAGCGCAGCATAACCACCATC; down: AGCCATTCCCTTTAGACTTG

TSLP up: TCAGGAGCCTCTTCATCCTG; down: TGGTCATTGAGGGCTTCTCT

18S up: GCAATTATTCCCCATGAACG; down: GGCCTCTCTAAACCATCCAA

**Histology, immunohistochemistry, immunofluorescence and immunoprecipitation.** Samples were embedded in OCT compound and frozen (skin, spleen) or fixed with buffered 4%PFA at 4ºC and embedded in paraffin (skin and bones). Skin sections (4 μm) were stained with hematoxylin and eosin, toluidine blue and Goldner’s Trichrome using standard methods or processed for immunoreactions. Sections were blocked in 1% BSA in PBS or in 1% BSA in TBS‑T for Phospho‑NF‑kB‑p65 for 45 minutes. Primary antibodies used were: polyclonal mouse anti‑Ki67 (NCL‑L‑KI67‑MM1, Novocastra, dil. 1/100); anti‑K14 (PRB‑155P, Covance, dil. 1/1000), anti‑K1 (PRB‑165P, Covance, dil. 1/500), anti‑Loricrin (PRB‑145P, Covance, dil. 1/500), anti‑TSLP (AF555 R&D Systems, dil 1/200 from 100ug/ml stock), anti‑Phospho‑NF‑kB‑p65 (Ser276) (3037, Cell Signaling Technology, dil. 1/50), anti‑CD31 (RM5200, Caltag, dil. 1/100), biotinylated anti‑B220 or biotinylated anti‑CD11b, mouse anti-LEKTI, (ZYMED Laboratories, 39-0500), anti-Desmoglein (dsg1, H-290) (Santa Cruz Biotechnology, sc-20114). Sections were then stained with the following secondary antibodies: anti‑mouse IgG HRP (P0447, Dako Switzerland AG, dil. 1/200), anti‑rabbit HRP (NA9340, Amersham, dil. 1/200), EnVision+ System-HRP (DAB), DakoCytomation (K4006, Mouse and K4010, Rabbit) or anti‑goat Alexa 488 (A‑11055, Molecular Probes, Invitrogen, dil. 1/200), anti‑rat alexa 568 (A11077, Molecular Probes, dil. 1/400) or streptavidin Alexa 488 (40462A, Molecular Probes, dil. 1/500). For immunochemistry, the slides were counterstained with Meyer’s hematoxylin, dehydrated and mounted with Eukitt Solution (O. Kindler GmbH & Co). For immunofluorescence, nuclei were stained with DAPI (D‑9542, Sigma). The sections were mounted with DABCO (D‑2522, Sigma). For immunoprecipitation two different Notch antibodies were used: ab8925 (Abcam) for activated Notch1, and C-20 (Santa Cruz Biotechnology) for C-terminus of Notch1.

**Skin Grafting.** Athymic *nu/nu* mice were prepared 3 weeks prior to engraftment by surgically implanting a 2cm glass coin under the back skin. Backskins were dissected from a total of 6 WT and N1N2K5 newborn mice, spread on sterile culture dishes with PBS and stored briefly at 4°C. During this time, each skin graft recipient site was prepared by removal of a patch of full thickness skin and the implanted coin. Donor skin was then placed on the graft bed and sewed on with Vicryl**Plus* 6‑0 thread (Johnson‑Johnson Int). Mice were kept on buprenorphine for 5 days. 2 months after transplantation, host mice were injected with tamoxifen as previously described.

**Bone marrow transplantation.** Bone marrow chimeras were generated using T cell–depleted bone marrow cells from RBP‑JK5 or C57BL/6L CD45.1+ mice. Ten‑week‑old RBP‑JK5 or C57BL/6L CD45.1+ mice were given 1000 rads of γ‑irradiation 24 hr prior to receiving 3 × 10^6^ donor bone marrow cells intravenously. Radiation chimeras were maintained on antibiotic water (Bactrim®, Roche) and analyzed after 2‑3 months.

**Analysis of cytokine and IgE production by ELISA.** Blood samples were collected by intracardiac punction, allowed to clot overnight at 2‑8°C before centrifugation at 2000 rpm for 30 minutes, frozen at ‑80°C and analyzed within 3 months with protein ELISA kits for IL‑1β, TNFα, G‑CSF and TSLP (all from R&D Systems) and for IgE levels (Bethyl Laboratories). Human venous blood samples were collected in Monovette serum collection tubes (Sarstedt) and centrifuged at 3000 rpm (1830 rcf) for 10 minutes. The serum was then aliquoted and frozen at -20°C before analysis by human TSLP ELISA kit (R&D Systems).

**Dermal and epidermal cell suspensions and CD45 cell MACS enrichment**. Ears from control and N1N2K5 mice were aseptically dissected and mechanically split into dorsal and ventral sides, and placed in 0.5% trypsin (Life Technologies) in 1xPBS containing 5% FCS. The tissue was digested for 30min at 37°C, the epidermis was separated from dermis as a single sheet and epidermal cell suspensions were obtained by filtering the trypsinized epidermal sheets through a stainless-steel sieve. Subsequently, the dermis was incubated for 2.5hr at 37°C in DMEM containing FCS (5%), CollagenaseD (Roche, 1.6mg/ml) and DNAse I (Roche, 40μg/ml) under continuous agitation. A single dermal cell suspension was obtained by filtering the digested dermis through a stainless-steel sieve. Dermal and epidermal cell suspensions were labelled with anti-CD45-FITC, incubated with anti-FITC magnetic beads and purified using a MACS separator following the instruction manual (Miltenyi Biotec).

**mAbs and flow cytometry.** The following antibodies were used: CD45-FITC (clone LCA, Caltag), CD3-PE (clone 17A2, BD Biosciences), CD19-PECy5.5 (clone 6D5, eBiosciences), CD11b-PECy7 (clone M1/70, eBiosciences), CD11c-PE (clone N418, eBiosciences), Vγ3-FITC (clone 536, BD Biosciences), Ly6G-PE (clone 1A8, BD Biosciences), PDCA-1-PE (clone eBio927, eBiosciences), Ly6C-biotinylated (clone HK1.4, Southern), CD103-biotinylated (clone 2E7, eBiosciences). Biotinylated mAbs were revealed with streptavidin-allophycocyanin. Flow cytometry data were acquired on a FACSCalibur flow cytometer using CellQuest software (BD Biosciences), and analyzed using FlowJo software (Tree Star). Dead cells were gated out by their forward and side scatter profile.

**Reference**

1. Perez-Moreno M, Fuchs E (2006) Catenins: keeping cells from getting their signals crossed. Dev Cell 11: 601-612.
