# Supplementary figures and images for: Atopic Dermatitis-Like Disease and Associated Lethal Myeloproliferative Disorder Arise from Loss of Notch Signaling in the Murine Skin
Source: PLoS One. 2010 Feb 18;5(2):e9258. doi: 10.1371/journal.pone.0009258 (PMC2823782; doi:10.1371/journal.pone.0009258)

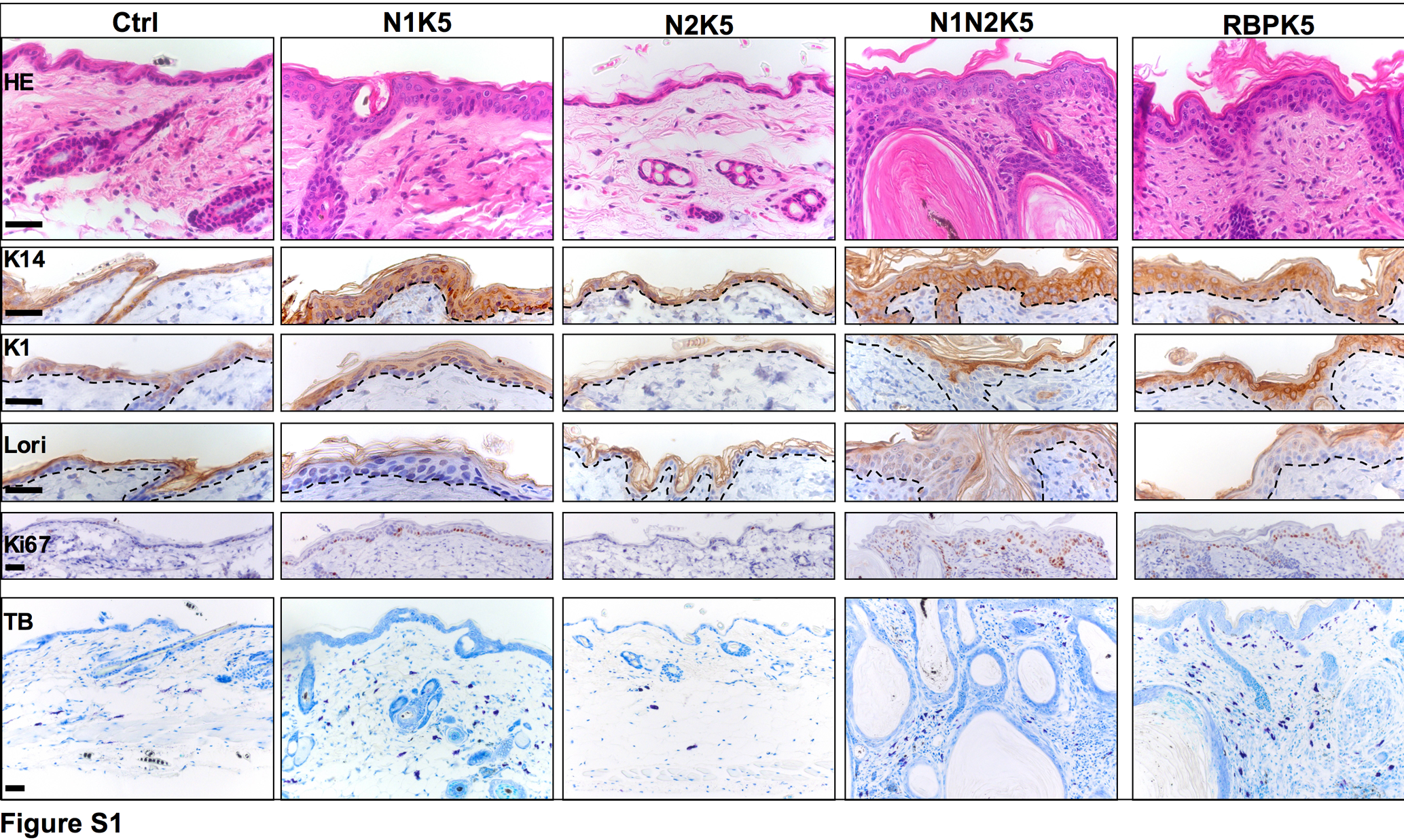

Supplement: Figure S1 — Loss of both Notch1 and Notch2, or RBP-J, but not Notch1 or Notch2 alone, leads to the development of a severe skin phenotype. Representative HE staining, Keratin14 (K14), Keratin1 (K1), Loricrin (Lori) and Ki67 immunohistochemistry and Toluidine Blue (TB) staining on dorsal skin sections from control (Ctrl), N1K5, N2K5, N1N2K5 and RBP-JK5 mice (all within 30-40 days of tamoxifen injection) (n = 8, from 4 individual experiments). Mice lacking Notch2 in the epidermis are indistinguishable from controls, whereas Notch1 deficient mice show K14 expression throughout the epidermis (whereas it is confined to the basal layer in controls), have a hyperproliferative epidermis and a mild increase in toluidine blue positive mast cells. Loss of Notch signaling in N1N2K5 or RBP-JK5 mice leads to an even more hyperproliferative phenotype with Ki67 positive cells throughout the entire epidermis, which is also K14 positive. However, K1 and Loricrin are still expressed in the upper layers of the epidermis. Loss of Notch signaling also leads to a massive dermal infiltration of mast cells [Scale bars: 50 µm]. (4.69 MB TIF) [file pone.0009258.s002.tif]

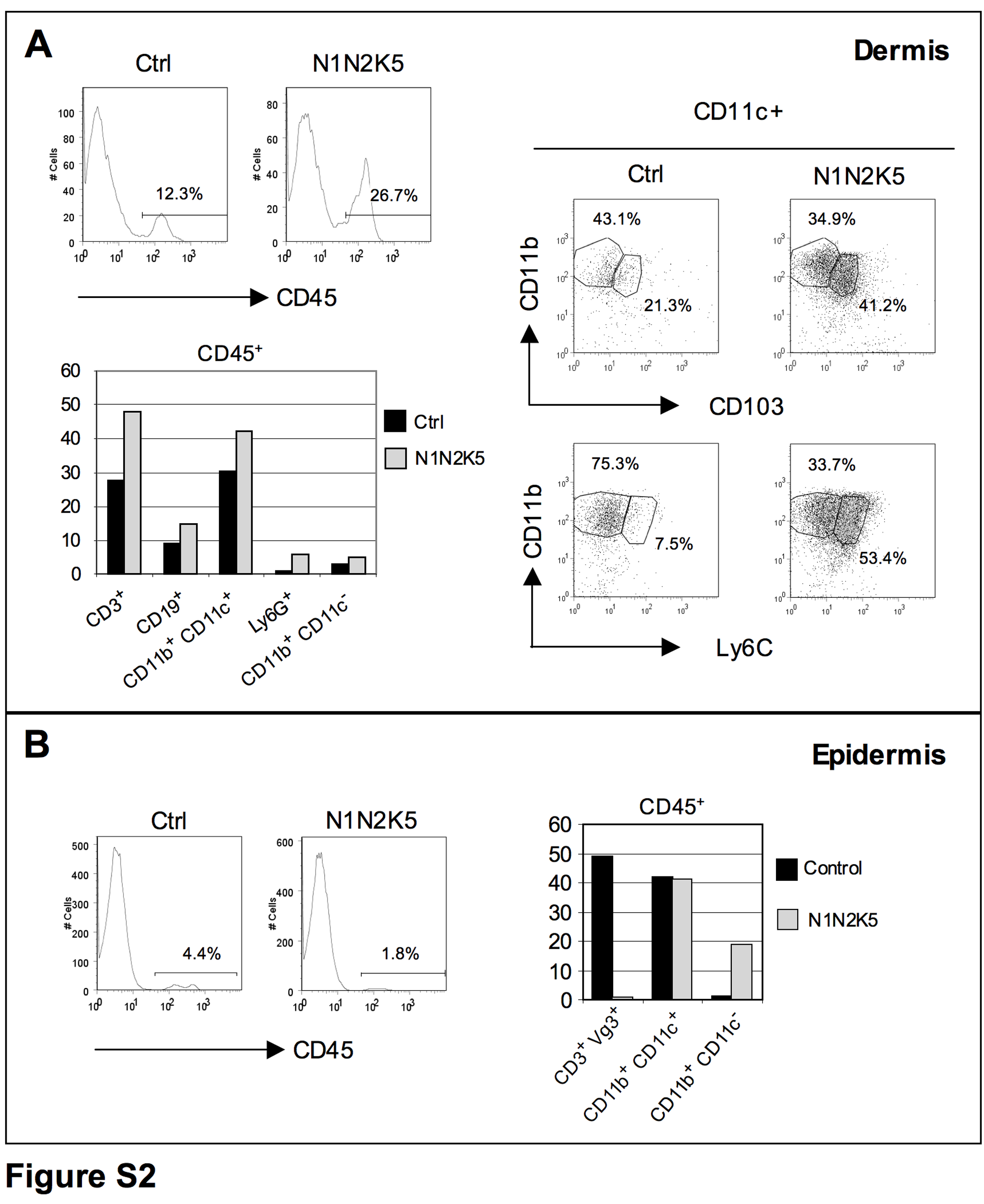

Supplement: Figure S2 — Analysis of DCs and other bone marrow derived cells in the skin. (A). Histograms show the staining of CD45 in total dermal cell suspensions from control (Ctrl) or N1N2K5 mice. The percentage of CD45+ cells is indicated. The bar diagram indicates the percentage of T cells (CD3+ cells), B cells (CD19+ cells), dendritic cells (DCs, CD11b+ CD11c+ cells), neutrophils (Ly6G+ cells) and other myeloid cells (CD11b+ CD11c-) within the CD45+ gate in control (Ctrl) or N1N2K5 dermis. Dot plots show analysis of DCs (gated as CD45+ CD11c+) populations in the dermis. CD11b versus CD103 staining displays an increase of dermal Langerin+ DCs (CD11b+ CD103+) in N1N2K5 mice (41.2%) compared to control (Ctrl) mice (21.3%). CD11b versus Ly6C staining shows an increase of inflammatory DCs (CD11b+ LyC+) in N1N2K5 dermis (53.5%) compared to Ctrl dermis (7.5%). Plasmacytoid DCs (CD11b- CD11c+ PDCA1+) were not detected either in control (Ctrl) or N1N2K5 dermis (data not shown). Prior to flow cytometric analysis the CD45 population as well as the populations of DCs were enriched using magnetic MACS cell separation. (B) Histograms show CD45 staining in total epidermal cell suspensions from control (Ctrl) and N1N2K5 mice. The percentage of CD45+ cells is indicated. The bar diagram indicates the relative percentage of murine dendritic epidermal T cells (DETCs, CD3+ Vγ3+), Langerhans cells (LCs, CD11b+ CD11c+), and CD11b+ CD11c- cells within the CD45+ gate in control and N1N2K5 epidermis. Flow cytometric analysis was performed after magnetic MACS cell enrichment. Cells analyzed were a pooled sample size of n = 8 for control or n = 8 for N1N2K5 from two individual experiments. (0.83 MB TIF) [file pone.0009258.s003.tif]

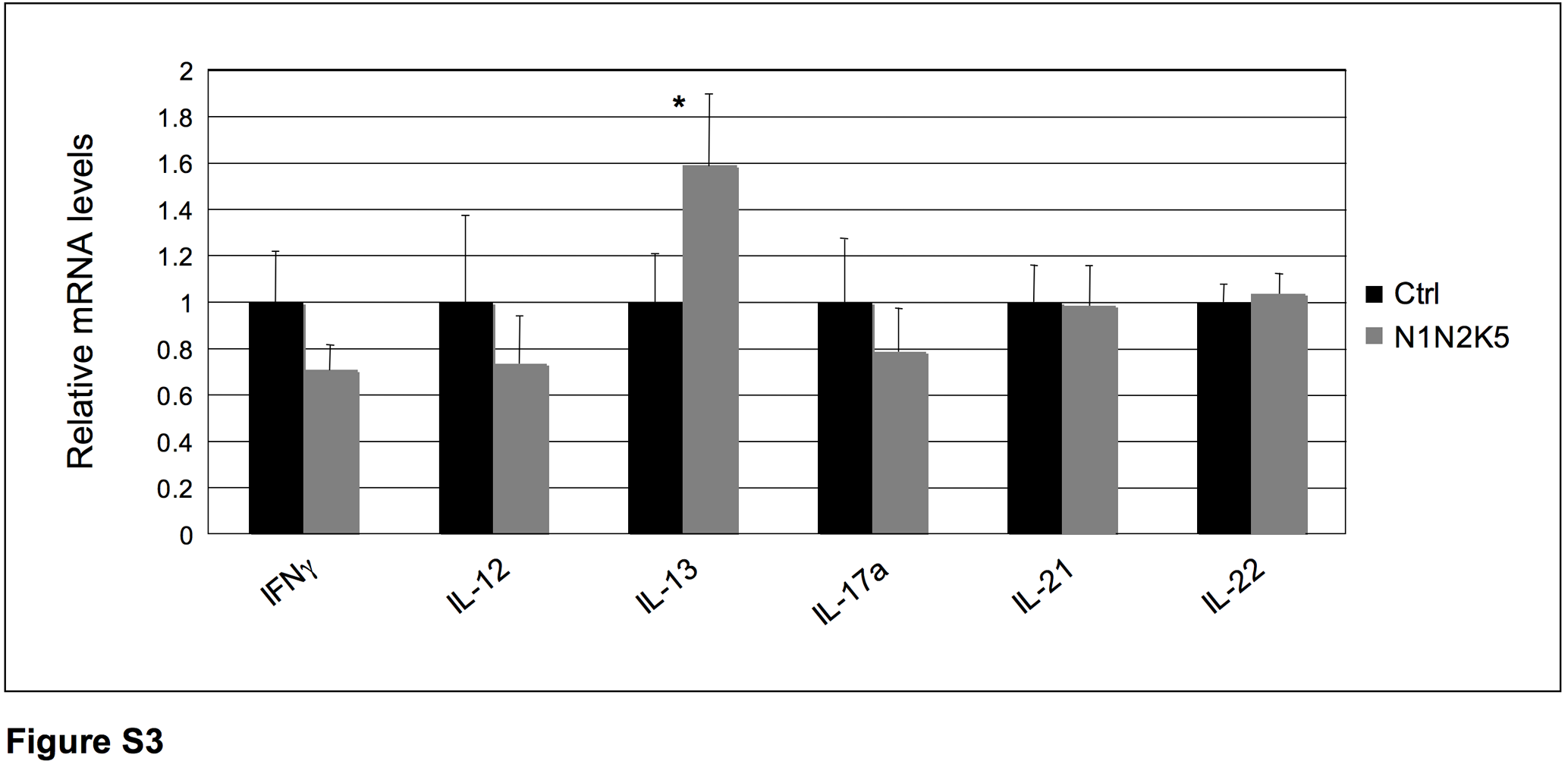

Supplement: Figure S3 — Quantitative RT-PCR on dermis-derived RNA for the T helper specific cytokines IFNγ, IL-12, IL-13, IL-17a, IL-21, IL-22 from Ctrl and N1N2K5 mice. Two to four individual experiments were performed (n = 3 per sample group); each experiment was run in triplicates (* p<0.01). (0.19 MB TIF) [file pone.0009258.s004.tif]

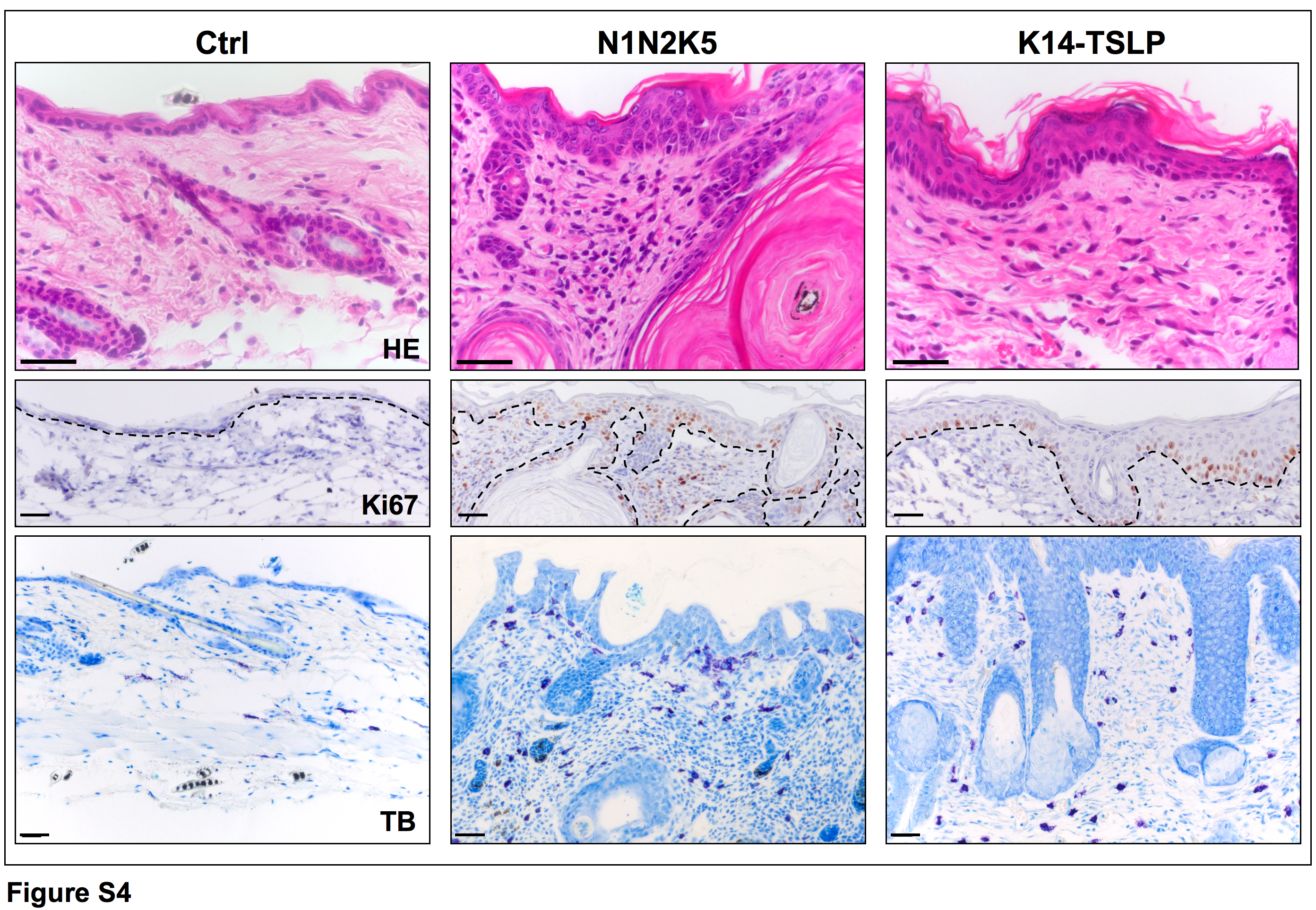

Supplement: Figure S4 — Skin-specific transgenic TSLP expression is sufficient to induce a similar AD phenotype as the Notch signaling deficient mice. Representative HE staining, Ki67 immunohistochemistry and Toluidine Blue (TB) staining on skin sections from control (Ctrl, n = 4), N1N2K5 (n = 8) and K14-TSLP mice (n = 4). The results shown are representative of 2 individual experiments. Both N1N2K5 and K14-TSLP mice show acanthosis, hyperkeratosis as well as dermal infiltration by mast cells [Scale bars: 50 µm]. (8.68 MB TIF) [file pone.0009258.s005.tif]

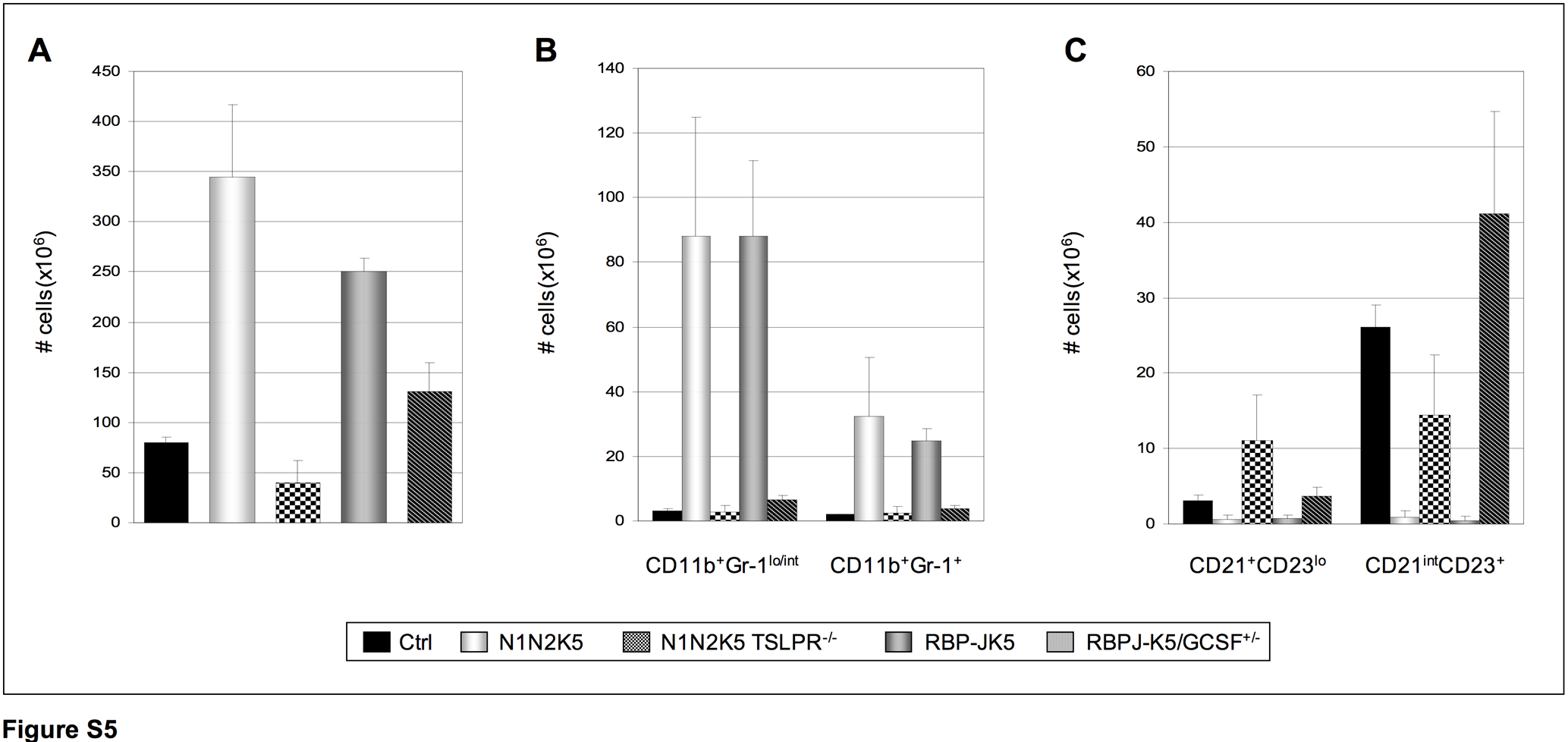

Supplement: Figure S5 — Loss of TSLP signaling or reduction of GCSF levels rescues the MPD. (A) Absolute cell numbers in the spleen of control, N1N2K5, N1N2K5 TSLPR−/−, RPB-JK5 and RBP-JK5/GCSF+/− mice. (B) Splenic absolute cell numbers of immature (CD11b+Gr.1lo/int) and mature (CD11b+Gr.1lo/int) myeloid populations, as well as (C) marginal zone (B220+CD21+CD23lo) and follicular (B220+CD21intCD23+) B cells. (n = 5 per sample group from two individual experiments). (0.42 MB TIF) [file pone.0009258.s006.tif]

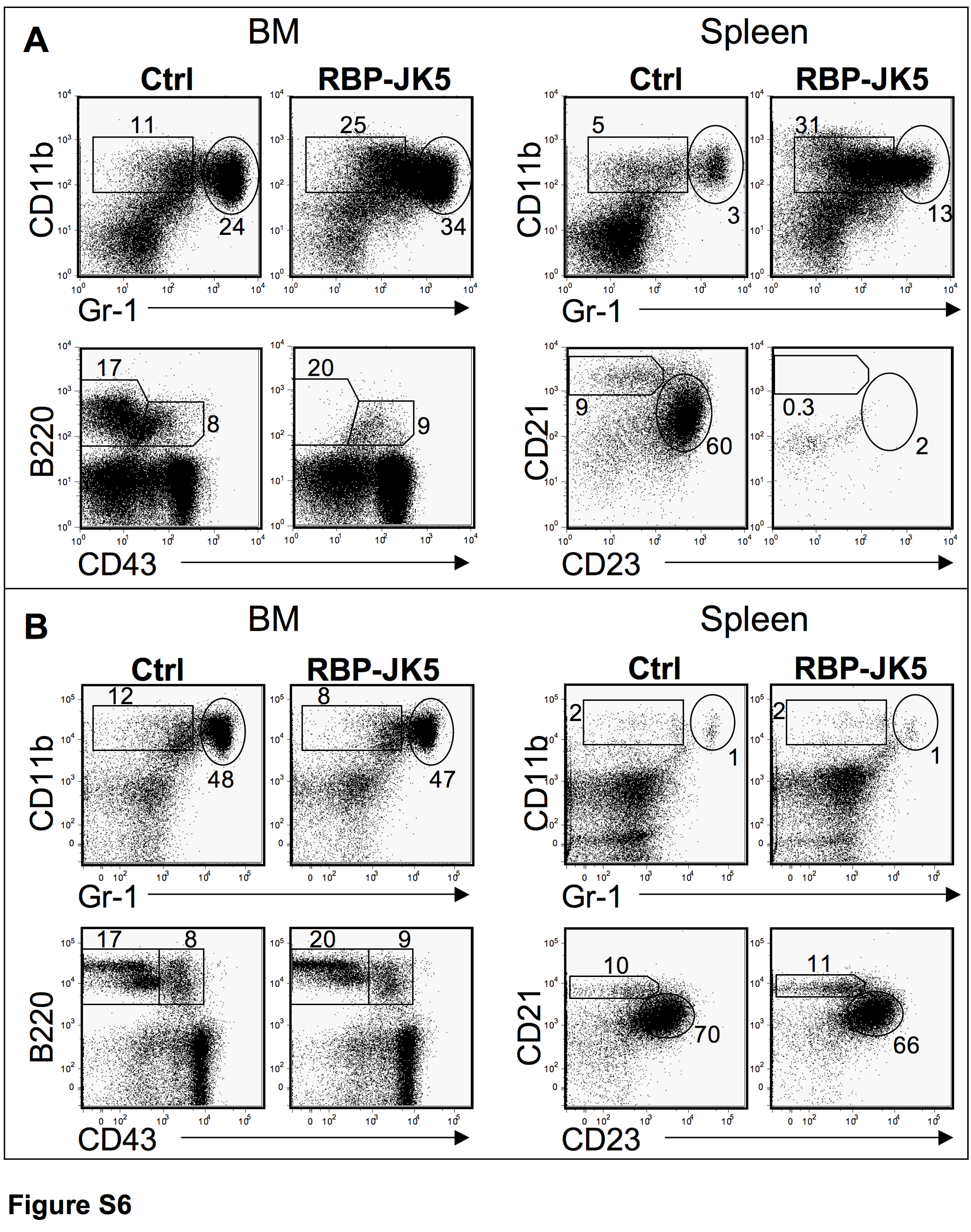

Supplement: Figure S6 — The MPD in mice lacking Notch signaling in the epidermis is cell non-autonomous. (A) Representative flow cytometric analysis of chimeras (WT CD45.1+ BM cells derived from n = 3 mice were transplanted into 9 lethally irradiated RBP-JK5 mice). WT cells adopt a similar myeloproliferative phenotype in the BM and spleen as in the RBP-JK5 mice. (B) Representative flow cytometric analysis of reverse chimeras (CD45.2+ BM cells derived from 3 sick RBP-JK5 mice were intravenously transplanted into 9 lethally irradiated WT CD45.1+ recipients). RBP-JK5 derived BM cells show normal hematopoiesis in a WT environment. The results in A and B are derived from 3 individual experiments. (2.40 MB TIF) [file pone.0009258.s007.tif]

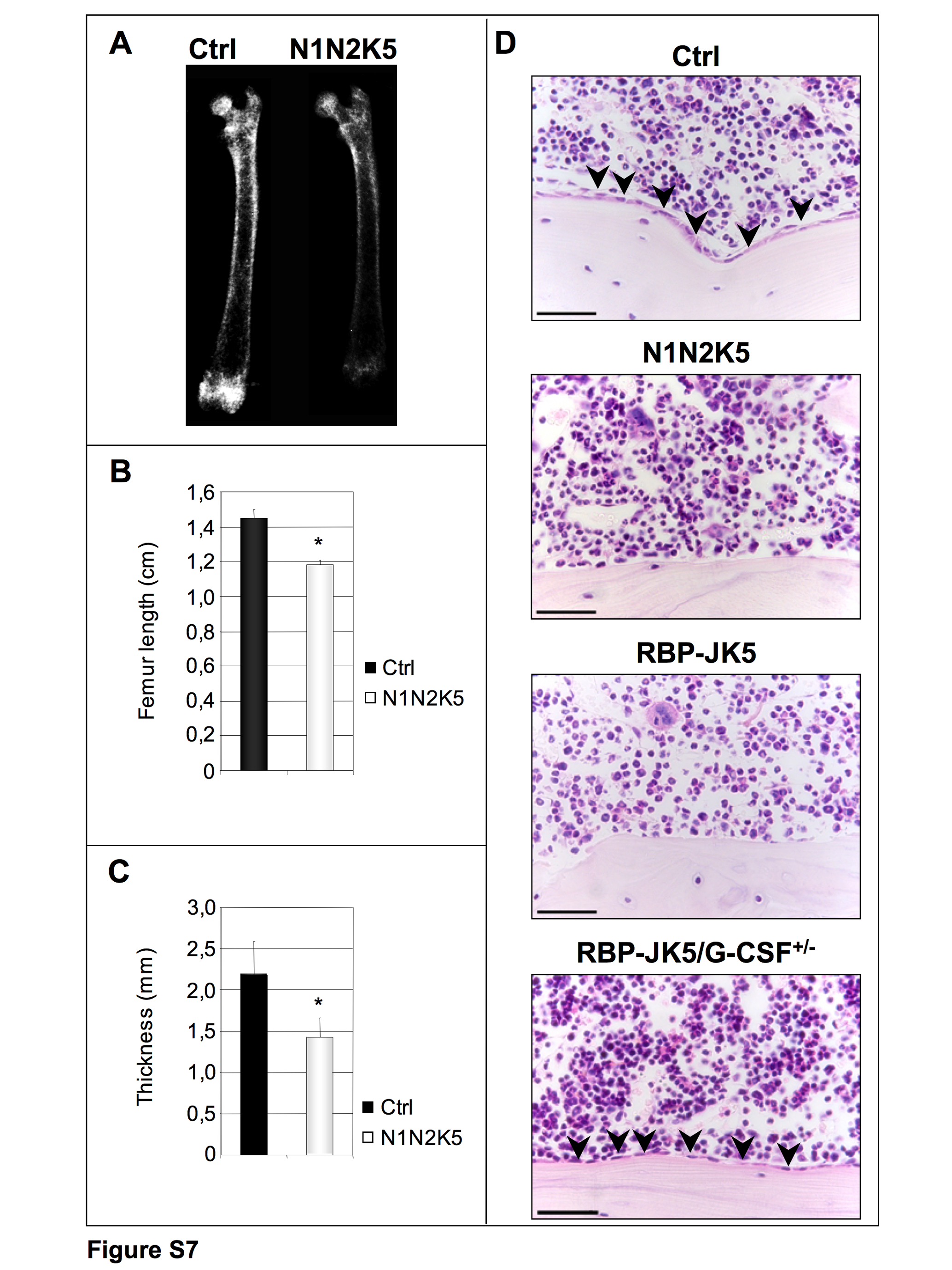

Supplement: Figure S7 — Loss of Notch signaling in the epidermis leads to osteopenia and is due to high G-CSF levels. (A) Representative X-ray analysis of femurs from control (Ctrl) and N1N2K5 mice showing a marked decrease in bone density in the mutant bone. (B) Femur length of control (Ctrl) and N1N2K5 mice (* = p value<0.05, n = 10, three individual experiments). (C) Femur cortical thickness of control (Ctrl) and N1N2K5 mice (* = p value<0.001, n = 10, three individual experiments). (D) Representative HE staining on femoral bone sections from control (Ctrl), N1N2K5, RBP-JK5 and RBP-JK5/G-CSF+/− mice showing a loss of endosteal cells in N1N2K5 and RBP JK5 mice and rescue of this phenotype in the RBP-JK5/G-CSF+/− mice (n = 5 per sample group, two individual experiments). [Scale bars: 50 µm]. (3.62 MB TIF) [file pone.0009258.s008.tif]

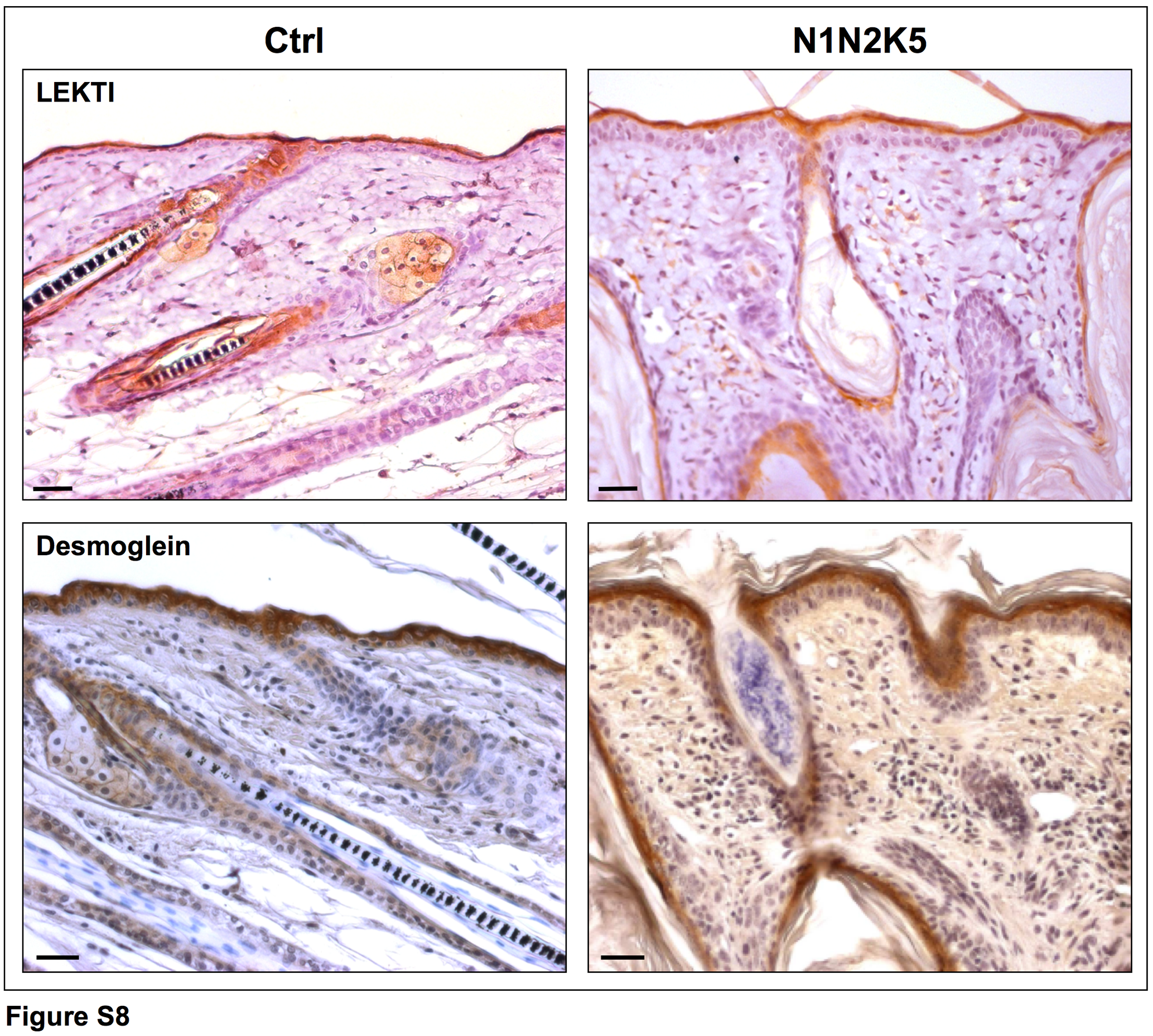

Supplement: Figure S8 — Intact stratum corneum in N1N2K5 mice. Down regulation or loss of LEKTI is frequently observed in skin with altered desquamation, impaired keratinization and skin barrier defects. LEKTI deficiency causes abnormal desmosome cleavage in the upper granular layer through degradation of desmoglein 1. This leads to defective stratum corneum adhesion and thus to the loss of barrier function. Positive staining for (A) the multi-domain serine protease inhibitor LEKTI (lympho epithelial kazal-type inhibitor) and (B) Desmoglein indicates the presence of an intact epidermis in both control (Ctrl) and N1N2K5 mice 5 weeks after gene inactivation (n = 3 mice per sample group, two individual experiments). [Scale bars: 50 µm]. (5.28 MB TIF) [file pone.0009258.s009.tif]

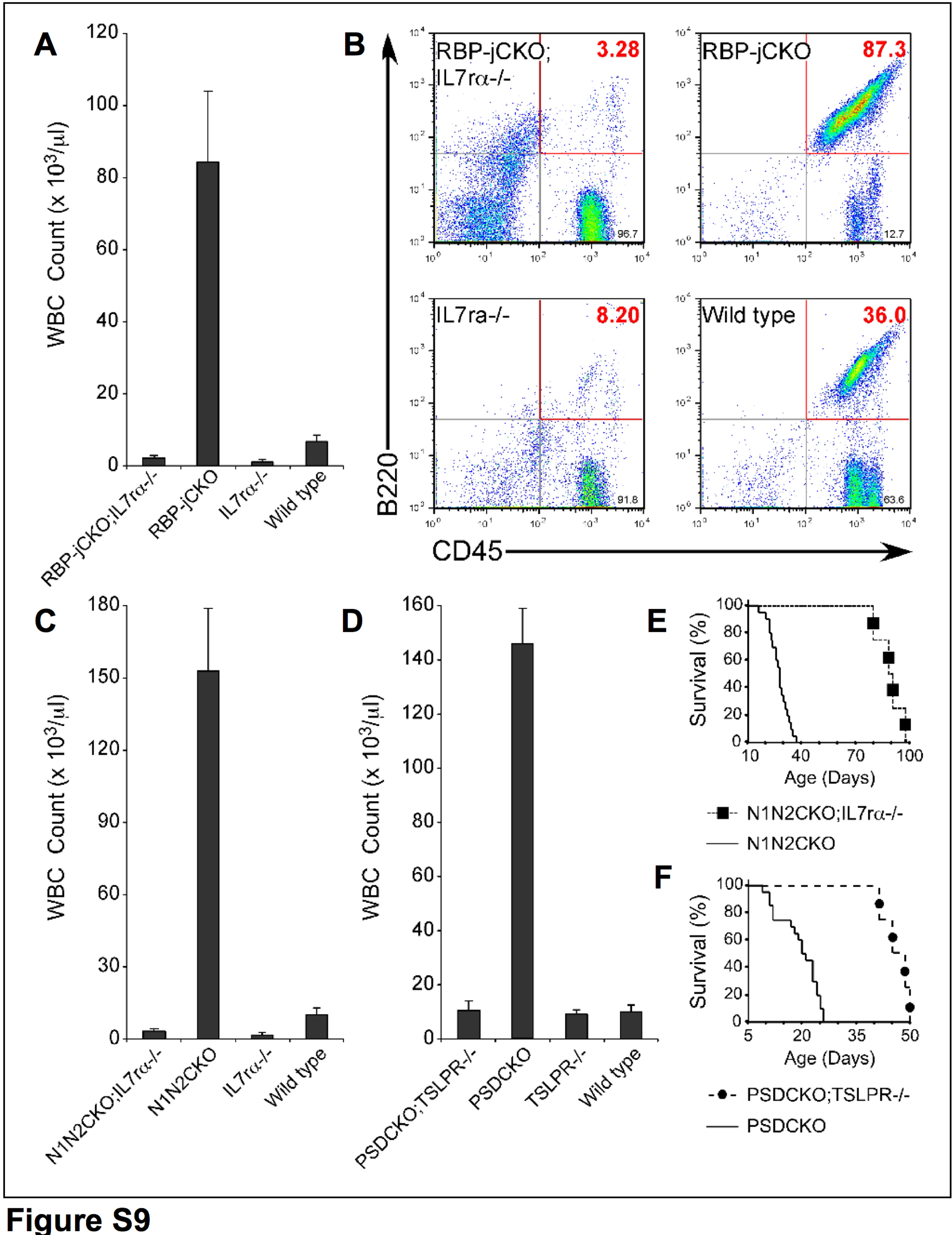

Supplement: Figure S9 — TSLP is required for B-LPD occurrence in Notch signaling-deficient animals. TSLP binds to a heterodimeric receptor that shares one of its subunits (IL-7rα) with IL-7 receptor. Inhibiting TSLP effects by removing either TSLPR (TSLPR−/−) or IL-7rα (IL7rα−/−) subunit of TSLP receptor leads to disappearance of B-LPD in the mutant mice. (A) WBC counts of RBP-j-deficient animals lacking IL-7rα (Msx2-Cre/+; RBP-jflox/flox; IL-7rα−/− or RBP-jCKO;IL7rα−/−) are within the normal range at P14. (B) Flow cytometric analysis on peripheral blood shows no signs of B-LPD in RBP-jCKO;IL7rα−/− mice compared to their Msx2-Cre/+; RBP-jflox/flox; IL-7Rα+/− (RBP-jCKO) littermates at P14. Representative results are shown with B220+ B cell percentage (red) in the upper right corner of each dot plot. IL-7rα−/− and wild type littermates are also analyzed as controls. Note that WBC counts and B cell percentage of RBP-jCKO;IL7rα−/− and IL-7rα−/− mice are lower than wild-type (IL-7rα+/−) littermates. This may be due to a simultaneous inhibition of IL-7 reception and reduction in baseline B and T cells especially in adulthood. (C) WBC counts of N1N2CKO animals lacking IL-7rα (Msx2-Cre/+; Notch1flox/flox; Notch2flox/flox; IL-7rα−/− or N1N2CKO;IL7rα−/−) are normal at P14. (D) Similar normalization of WBC counts is seen with PSDCKO animals that have lost the TSLPR arm of TSLP receptor (Msx2-Cre/+; PS1flox/flox; PS2flox/flox; TSLPR−/− or PSDCKO;TSLPR−/−). (E, F) B-LPD prevention leads to increased life span among (E) N1N2CKO;IL7rα−/− and (F) PSDCKO;TSLPR−/− animals compared to their N1N2CKO and PSDCKO littermates respectively (p<0.001, log rank test). In each panel, data are compiled from 4 animals in each group from two individual experiments. (1.75 MB TIF) [file pone.0009258.s010.tif]
